# Palladium-catalyzed asymmetric three-component reaction between glyoxylic acid, sulfonamides and arylboronic acids for the synthesis of *α*-arylglycine derivatives

**DOI:** 10.3389/fchem.2023.1165618

**Published:** 2023-03-13

**Authors:** Bastian Jakob, Andreas M. Diehl, Kathrin Horst, Harald Kelm, Georg Manolikakes

**Affiliations:** RPTU Kaiserslautern-Landau Organic Chemistry, Kaiserslautern, Germany

**Keywords:** palladium-catalysis, asymmetric catalysis, amino acids, multicomponent reaction, petasis reaction, sulfonamides, boronic acids

## Abstract

A palladium-catalyzed asymmetric three-component synthesis of *α*-arylglycine derivatives starting from glyoxylic acid, sulfonamides and arylboronic acids is reported. This novel, operationally simple method offers access to the *α*-arylglycine scaffold in good yields and enantioselectivities. The utilization of α tailored catalyst system enables the enantioselective synthesis of the desired *α*-arylglycines despite a fast racemic background reaction. The obtained products can be directly employed as building blocks in peptide synthesis.

## 1 Introduction

**FIGURE 1:**
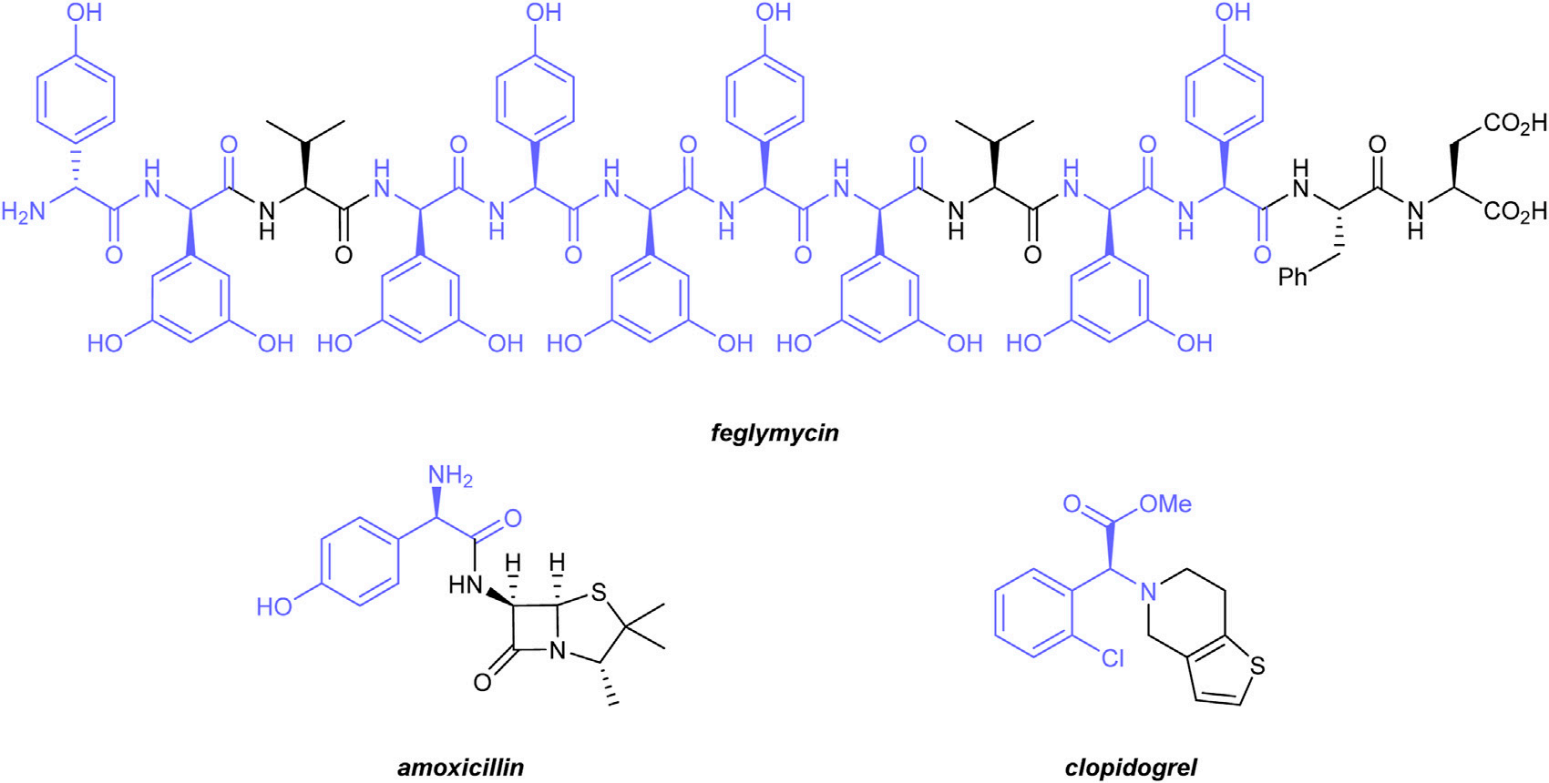
Biologically active molecules containing *α*-arylglycine motifs (highlighted in blue).

α-Amino acids play a central role in biology and chemistry. As the smallest unit of all peptides and proteins, they are the building blocks of life (Nelson and Cox, 2008). *α*-Amino acids are important intermediates in the chemical industry and are used for the production of drugs, agrochemicals, or functional materials (Hughes, 2011). Due to the tremendous advances in the development of protein-based drugs (Tsomaia, 2015) and protein engineering (Lutz, 2010), unnatural and non-proteinogenic amino acids are gaining increasing importance. Within this class, *α*-arylglycines are of particular significance. The *α*-arylglycine motif is present in several natural products with unique biological activities. Prominent examples include vancomycin or teicoplanin, two glycopeptide antibiotics (Van Bameke et al., 2004), or Feglymycin (Dettner et al., 2009) (Figure 1), a 13-mer peptide containing nine *α*-arylglycine units, which shows promising activities against HIV and methicillin-resistant *Staphylococcus aureus* (MRSA). *α*-Arylglycines are useful building blocks for the synthesis of various drugs, such as amoxicillin (Fisher and Mobashery, 2014), an *α*-lactam antibiotic, or clopidogrel, an antiplatelet medication (Plosker and Lyseng-Williamson, 2007) (Figure 1). In addition, *α*-arylglycines are widely used as starting materials for the preparation of chiral auxiliaries or ligands (Hughes, 2011).

Due to their chemical and biological significance, various different methods for the asymmetric synthesis of *α*-arylglycine derivatives have been developed (Nájera and Sansano, 2007; Hughes, 2011). Multicomponent reactions (MCR) based on the *in-situ* generation of reactive imine species offer a particularly attractive approach toward the *a*-arylglycine scaffold (Herrera and Marqués-López, 2015).

**SCHEME 1:**
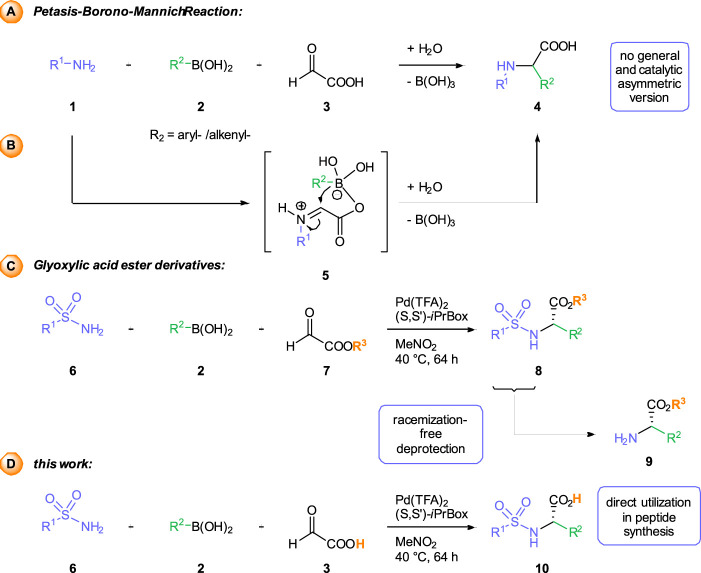
Synthesis of α-arylglycines *via* the racemic Petasis reaction **(A)**. Previous work based on the usage of glyoxylates for enantiomeric synthesis **(B)** and this work with the free glyoxylic acid **(C)**.

Among these different MCRs, the Petasis or borono-Mannich reaction (Scheme 1A), is ideally suited for the construction of arylglycine derivatives due to the simple and widespread availability of the starting materials (Candejas et al., 2010; Paul, Presset and Le Gall, 2017; Wu, Givskov and Nielsen, 2019). In this three-component process an amine 1) and an aryl or alkenyl boronic 2) react with glyoxylic acid 3) in the absence of any catalysts to furnish *a*-arylglycines 4) with a high degree of structural diversity. Mechanistic studies support a reaction pathway involving an additional activation of the boronic acid as ate complex five and intramolecular transfer of the organic residue (Scheme 1B). As the Petasis reaction proceeds in the absence of any catalyst, it gives access to racemic amines. Examples of enantioselective Petasis reactions are rare and often limited to specific substrate combinations (Lou and Schaus, 2008). (Dia) Stereoselective construction of the newly formed stereocenter is usually achieved by using chiral amine or aldehyde components.

Recently, we have disclosed palladium-catalyzed, three-component transformations between (sulfon) amides, different aldehyde components and boronic acids or carboxylic acids as broadly applicable alternatives to the classical Petasis reaction (Beisel and Manolikakes. 2013; Beisel and Manolikakes, 2015; Beisel, Diehl and Manolikakes, 2016; Beisel and Manolikakes, 2016; Diehl and Manolikakes, 2020). With glyoxylic acid ester as aldehyde component, the corresponding *a*-arylglycines esters 8) can be obtained in high yields and enantioselectivities (scheme 1c) (Beisel, Diehl and Manolikakes, 2016). Incorporation of 2, 2, 4, 6, 7-pentamethyl-2, 3-dihydrobenzofuran-5-sulfonylamide (Pbf-NH_2_) enables the synthesis of Pbf-protected arylglycines. Racemization-free cleavage of the Pbf-group affords the glycine derivatives 9) with a free N-terminus for further transformations, such as peptide coupling. On the other hand, hydrolysis of the ester group in obtained arylglycine derivatives leads to (partially) racemized products (Beisel, Diehl and Manolikakes, 2016; Tailhades, 2022). An enantioselective approach to *a*-arylglycine derivatives 10), which can be easily modified both on the N- and the C-terminus, would be highly desirable. Such products would provide versatile building blocks for the synthesis of arylglycine-containing peptides or natural products.

Herein, we describe a palladium-catalyzed enantioselective three-component reaction between arylboronic acids, sulfonamides and the parent glyoxylic acid itself (Scheme 1D). Although the scope, yields and stereoselectivities of this novel transformation so far are only moderate, this process provides an alternative approach to the arylglycine derivatives, which can be directly utilized in peptide synthesis. The identification of a specifically tailored catalyst system, allowed us to override an otherwise fast racemic background reaction.

## 2 Results and discussion

**SCHEME 2:**
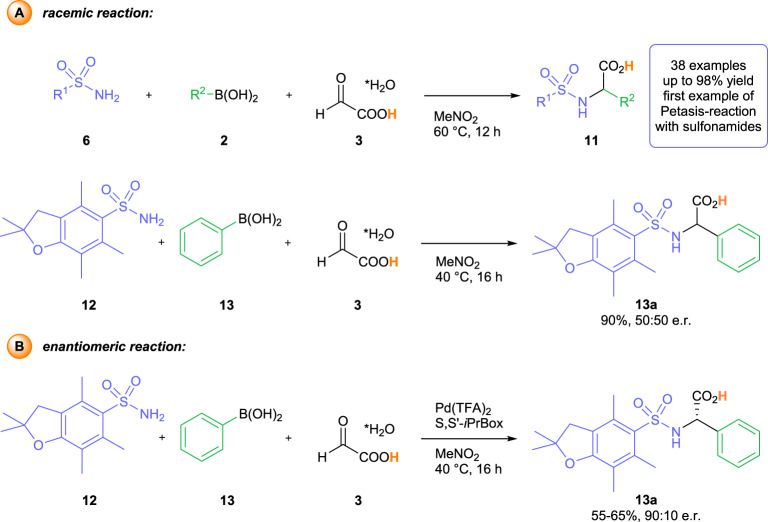
Racemic background reaction **(A)** and initial observations of the enantiomeric reaction **(B)**.

Initially, we performed a detailed analysis of potential amine and aldehyde components and protecting group combinations, which could provide access to arylglycine products with handles for a chemoselective modification of the N- and the C-terminus. Whereas the previously identified Pbf-protecting group is perfectly suited for this purpose (Carpino et al., 1993; Isidro-Llobet, Álvarez and Albericio, 2009), the ideal choice for the carboxylic acid part would be the free, unprotected glyoxylic acid itself. The thereby assembled arylglycine products bear close resemblance to Boc-protected amino acids, which are among the most common building blocks for (solid-phase) peptide synthesis (Isidro-Llobet, Álvarez and Albericio, 2009; El-Faham and Albericio, 2011). Yet, the direct utilization of free glyoxylic acid poses one fundamental problem for the development of an enantioselective transformation. Due to its free OH-group, glyoxylic acid is a perfect substrate for a classical Petasis reaction. Indeed, we have shown previously, that the reaction of glyoxylic acid with sulfonamides and aryl boronic acids furnished racemic α−arylglycines 11) in the absence of any catalyst or additive (Scheme 2A) (Diehl et al., 2018). As an example, the reaction of 2, 2, 4, 6, 7-pentamethyl-2, 3-dihydrobenzofuran-5-sulfonylamide 12) and phenylboronic acid 13) with glyoxylic acid, used in the easy-to-handle solid monohydrate form 3), furnishes the Pbf-protected phenylglycine 14) in 90% yield after 16 h at 40°C in nitromethane as solvent. First test reactions with our previously established Pd (TFA)_2_—*S,S′-iPr*Box-catalyst systems (Beisel and Manolikakes. 2013; Beisel and Manolikakes, 2015; Beisel, Diehl and Manolikakes, 2016; Beisel and Manolikakes, 2016; Diehl and Manolikakes, 2020) led to highly interesting observations. In the presence of the palladium catalyst, the desired *a*-arylglycine (13a) was obtained in a decreased yield of 55%–65% under otherwise identical reaction conditions (MeNO_2_, 40°C for 16 h) (Scheme 2B). To our surprise, we observed a high degree of enantioinduction in the presence of the chiral catalyst. The desired arylglycine could be isolated with an enantiomeric ratio (e. r.) of up to 90:10. However, we quickly encountered problems with reproducibility. Whereas the isolated yield stayed in a range of 55%–65% for several independent experiments, the enantiomeric ratio for individual runs ranged between 60:40 to 90:10.

Despite these inconsistencies, we were encouraged by the observed enantioselective formation of *a*-arylglycine 13a under conditions adapted from the racemic, catalyst-free Petasis reaction. Therefore, we started a more thorough examination of the role of solvents and additives in the palladium-catalyzed, enantioselective three-component reaction between sulfonamide 12, glyoxylic acid monohydrate three and phenylboronic acid 13 (Table 1). We quickly identified the crucial role of trace amounts of water in the outcome of the reaction. A reaction with pre-dried nitromethane and rigorous exclusion of ambient moisture afforded arylglycine 13a in 57% isolated yield and an e. r. Of 57:43. Increasing the amount of water from 1.0 to 5.0 equivalents led to a drastic increase in enantioselectivity with consistent isolated yields (entries 2–4). Further increase of the water content resulted in decreased yields and enantioselectivities (entry 5). The use of commercially available, aqueous glyoxylic acid solution (50 wt%; equals 6.6 equivalents of water) furnished arylglycine 13a in a lower yield of 34%, albeit with very high enantioselectivity. As arylboronic acids can contain varying amount of the corresponding boroxines, water might play an important role in liberating the free boronic acid as a crucial intermediate for the palladium-catalyzed reaction (Schrapel and Peters, 2015). Indeed, the reaction with triphenylboroxine afforded the arylglycine product 13a in only 34% yield and an enantiomeric ratio of 64:36 (entry 7). Still, one has to consider, that during the reaction system up to two equivalents of water are generated over the curse of the reaction (one equivalent from the used glyoxylic acid hydrate and one from the condensation of glyoxylic acid with the sulfonamide). Overall, the addition of five equivalents of water led to the best results, both in terms of isolated yield and enantioselectivity. Therefore, the effect of further solvents, both with and without five equivalents of water as additive, were investigated. Reactions in chlorinated solvents, such as CH_2_Cl_2_ or CHCl_3_, furnished arylglycine 13a in 31%–82% yield and low to modest enantioselectivities (e. r. ≤ 65:35, entries 8–11). No significant effect of water was observed for these chlorinated solvents. Ether-based solvents, such as THF, 2-Me-THF, MTBE or 1, 4-dioxane resulted in overall lower yields of 3%–36% and very low enantioselectivities (entries 12–19). The addition of water had a detrimental effect on the isolated yield in all four cases. Only the use of anhydrous THF afforded the desired arylglycine in modest enantioselectivity (entry 11). No product formation could be observed in protic solvents, such as EtOH, MeOH or pure water (entry 20). In toluene and acetonitrile, the desired arylglycine was obtained in 61%–63% yield as a nearly racemic mixture (entries 21 and 23). Addition of water only resulted in decreased yields without a significant effect on the stereochemical outcome (entries 22 and 24).

**TABLE 1:** Initial experiments and solvent optimization.

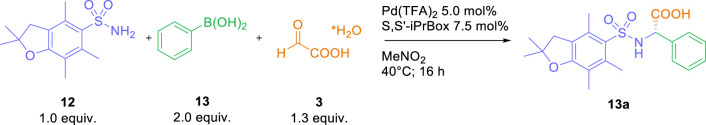			

Entry	Deviations from optimized conditions	Yield (%)^[a]^	e.r
1	Predried Nitromethane	57	57:43
2	1.0 eq H_2_O	65	89:11
3	2.5 eq H_2_O	59	93:7
4	5.0 eq H_2_O	54	97:3
5	10.0 eq H_2_O	39	74:26
6	Glyoxylic acid sol. (50wt%; 6.6 eq H_2_O)	34	99:1
7	Triphenylboroxine instead of 13	34	64:36
8	DCM	79	56:44
9	DCM +5.0 eq H_2_O	82	61:39
10	Chloroform	31	65:35
11	Chloroform +5.0 eq H_2_O	55	61:39
12	THF	36	72:28
13	THF +5.0 eq H_2_O	17	53:47
14	2-Me-THF	15	52:48
15	2-Me-THF +5.0 eq H_2_O	3	51:49
16	MTBE	14	51:49
17	MTBE +5.0 eq H_2_O	9	55:45
18	1,4-Dioxane	32	52:48
19	1,4-Dioxane +5.0 eq H_2_O	17	52:48
20	MeOH/EtOH or H_2_O	NR	-
21	Acetonitrile	61	52:48
22	Toluene	63	54:46
23	Acetonitrile +5.0 eq H_2_O	22	53:47
24	Toluene +5.0 eq H_2_O	23	51:49

[a]Isolated yield of analytically pure product. DCM, dichlormethane; THF, tetrahydrofuran; MeOH, methanol; EtOH, ethanol; NR, no reaction.

With these optimized conditions, we investigated the effect of different (bis)oxazoline-type ligands. The rather unexpected outcome is summarized in Table 2. Reactions with Box-ligands L2-L5, containing either a different spacer between the oxazoline-rings or residues with a different steric demand, afforded the arylglycine product 13a in 7%–55% yield and almost racemic form (entries 2–5). Similar stereoselectivities were obtained with the pyridine-derived ligands L6 and L7 (entries six and 7). Considering the common observation, that small changes in ligand structure usually result in small changes in observed yields/stereoselectivities (Ghosh, Mathivanan and Cappiello, 1998; Desimoni, Faita and Jorgensen, 2006; Desimoni, Faita and Jorgensen, 2011), the almost complete lack of stereoinduction for all other screened ligands seems rather surprising. We assume, that only ligand L1 displays the correct properties (bite-angle and steric demand) for a successful enantioselective three-component reaction.

**TABLE 2:** Variation of (bis)oxazoline-type ligands.

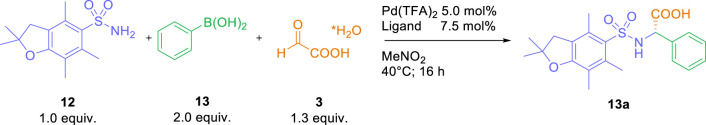			

Entry	Deviations from optimized conditions	Yield (%)^[a]^	e.r
L1	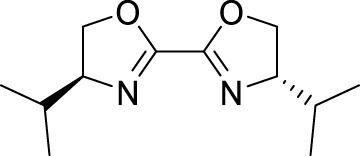	54	97:3
L2	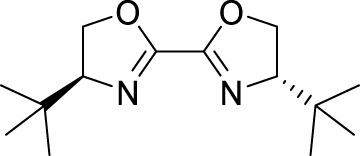	17	58:42
L3	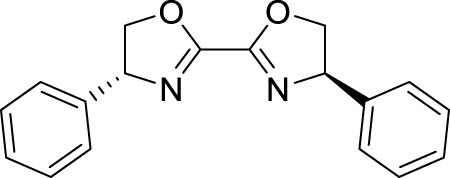	26	56:44
L4	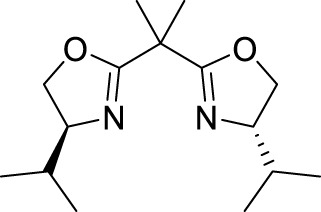	55	54:46
L5	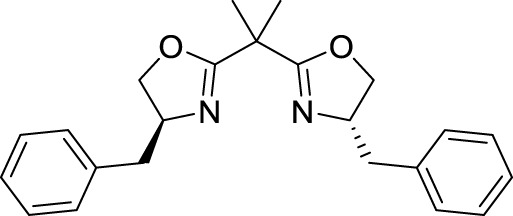	8	57:43
L6	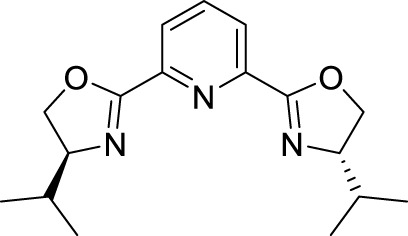	39	54:46
L7	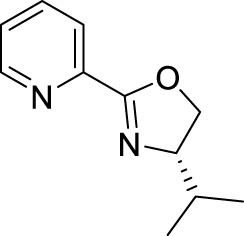	36	56:44

[a] Isolated yield of analytically pure product.

Next, we investigated the influence of different palladium precursors, catalyst loading and reaction temperature (Table 3). Decreasing the amount of Pd (TFA)_2_ to 2.5 or even 1 mol% furnished arylglycine 13a in approximately 50% yield with decreasing levels of enantioselectivity (entries two and 3). On the other hand, increasing the Pd (TFA)_2_ loading to 10 mol% afforded the desired arylglycine with an increased enantiomeric ratio of 99: 1, albeit with a consistent yield of 54% (entry 4). Alteration of reaction temperature showed a significant effect on yield and enantioselectivity (entries 5–7). A decreased temperature led to increased enantioselectivity together with a decreased yield (entry 5). Increased reaction temperatures afforded the arylglycine product in higher yield with significantly lower enantioselectivity (entries six and 7). With PdCl_2_ as a precursor, arylglycine 13a was obtained in 62% yield as a racemate (e. r. = 50:50, entry 8). The use of Pd(OAc)_2_ resulted in a similar yield (57%) together with a good level of stereoinduction (e. r. = 80:20, entry 9). These results indicate, that the pre-coordination of the ligand to Lewis-acidic palladium (II)-precursors might be crucial for the formation of an active catalyst species (Fanourakis et al., 2020). Indeed, pre-stirring of Pd (TFA)_2_ and L1 in MeNO_2_ for 30 min before adding all other starting materials led to the desired product with increased yield and enantioselectivity (entry 10). It is worth mentioning, that a slow erosion of the enantiomeric excess can occur over time. Whereas, the enantiomeric ratio decreased from 99:1 to 97:3 after a reaction time of 40 h (entry 11), a significant drop to 87: 13 was observed after 72 h (entry 12).

**TABLE 3:** Reaction parameter optimizations.

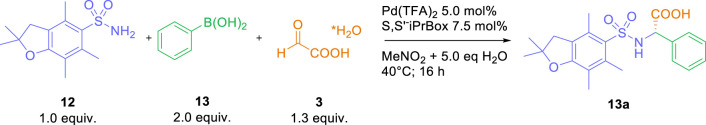			

Entry	Deviations from optimized conditions	Yield (%)^[a]^	e.r
1	no variation	54	97:3
2	1.0 mol% Pd (TFA)_2_	52	54:46
3	2.5 mol% Pd (TFA)_2_	33	75:25
4	10.0 mol% Pd (TFA)_2_	54	99:1
5	23°C instead of 40°C	23	96:4
6	50°C	59	76:24
7	60°C	70	71:29
8	PdCl_2_ instead of Pd (TFA)_2_	62	50:50
9	Pd(OAc)_2_ instead of Pd (TFA)_2_	57	80:20
10	Pd (TFA)_2_ and L1 prestirred for 30 min	64	99:1
11	Pd (TFA)_2_ and L1 prestirred for 30 min; 40 h reaction time	63	97:3
12	Pd (TFA)_2_ and L1 prestirred for 30 min; 72 h reaction time	61	87:13

[a] Isolated yield of analytically pure product.

With these optimized conditions, we investigated the three-component reaction of Pbf-sulfonamide 12 and glyoxylic acid hydrate 3) with different arylboronic acids 2) (Scheme 2). The reaction with *para*-tolyl boronic acid (14), *para*-fluorophenyl boronic acid (15) and *para*-chlorophenyl boronic acid (16), three arylboronic acids with similar electronic properties (Hansch, Leo and Taft, 1991), afforded the desired arylgylcines 14a-16a in 61%–74% yield and high enantioselectivities (e. r. 87:13–92:8). On the other hand, the more electron-rich 4-methoxyphenylbornic acid (17) furnished arylglycine 17a in 68% yield and a significantly lower enantiomeric excess of 60:40. For transformations with arylboronic acids bearing stronger electron-withdrawing groups, such as *meta*-chlorophenyl boronic acid (18), the product 18a was obtained in a lower yield of 47% yield and decreased enantioselectivity. Reaction with the sterically hindered methyl- and chloro-*ortho*-substituted arylboronic acids 19 and 20 delivered the desired *a*-arylglycines 19a and 20a in low yields and enantioselectivities. Overall, the electronic and steric properties of the employed boronic acid component display a significant influence on the outcome of the reaction, in particular enantioselectivity. This is in stark contrast to our previously reported catalyst-free and racemic transformation, which performs equally well with various substitution patterns (Diehl et al., 2018).

In order to evaluate the utility of the obtained Pbf-protected products as building blocks for peptide synthesis, arylglycine 13a was employed in the synthesis of a representative dipeptide. Coupling of 13a with l-valine methyl ester hydrochloride with HATU ((1-[Bis(dimethylamino) methylene]-1H-1,2,3-triazolo [4,5-*b*]pyridinium 3-oxide hexafluorophosphate) (Stetsenko et al., 2016) afforded the Pbf-protected dipeptide 22 in 90% yield. Removal of the Pbf-group was achieved with TFA in the presence of dimethyl sulfide, furnishing dipeptide 23 as the corresponding TFA salt in 95% yield. Importantly, no racemization was observed in the coupling or deprotection step. The final dipeptide 23 could be obtained in a diastereomeric ratio of 98:2 starting from an arylglycine batch with an enantiomeric ratio of 98:2. The successful preparation of the simple model dipeptide 23 model, showcases the utility of the Pbf-protected arylglycines as building blocks for peptide synthesis. Both, coupling of the protected building block and subsequent removal of the Pbf-group, could be achieved with standard procedures (El-Faham and Albericio, 2011; Stetsenko et al., 2016). One could envision, that the used procedures could be easily adapted to solid-phase peptide synthesis.

Finally, we also studied the incorporation of some selected sulfonamide residues into our three-component process (Scheme 5). Whereas the reaction with a simple sulfonamide, such as para-toluene sulfonamide 25, furnished the expected arylglycine product 25a in good yields and high enantioselectivities, no product formation was observed with the electron-poor para-nitrobenzene sulfonamide 26. Interestingly, a racemic product 27a was obtained with the sterically hindered sulfonamide 27. As the major focus of our work was devoted to the synthesis of a suitably protected arylglycine-building blocks for peptide synthesis, no further sulfonamides were studied.

In the first step, a condensation of the sulfonamide and glyoxylic acid to the corresponding imine **28** takes place. From imine 28, the mechanism bifurcates into two different pathways. In pathway A (uncatalyzed reaction) the boronic acid coordinates to the free hydroxy functionality of the glyoxylic acid, leading to the ate complex 29 (Candeias et al., 2010; Candejas et al., 2010; Paul, Presset and Le Gall, 2017; Wu, Givskov and Nielsen, 2019). Intramolecular transfer of the aryl moiety from the activated ate species to the electrophilic *N*-sulfonylimine leads to the addition product 30. Hydrolysis of 30 affords to final *a*-arylgylcine 11.

In competing pathway B (palladium-catalyzed reaction), a transmetallation from the boronic acid to the Lewis-acidic Pd(II)-complex 32 takes place (Dai and Lu, 2007; Dai, Yang and Lu, 2008; Ma, Zhang and Shi, 2009; Chen et al., 2012; Yang and Zhang, 2013; Schrapel and Peters, 2015; He et al., 2016; Yan et al., 2016; Wen et al., 2022). The stereoselective transfer of the aryl group from the Pd-complex to the imine generates intermediate 33. Product decomplexation from intermediate 33 leads to the *a*-arylgylcine product 10. In order to achieve a high degree of enantioselectivity the complete catalytic cycle of pathway B has to be significantly faster than the uncatalyzed reaction (pathway A). Factors, which slow down any individual step in the catalytic cycle should therefore lead to reduced enantioselectivities.

**SCHEME 3:**
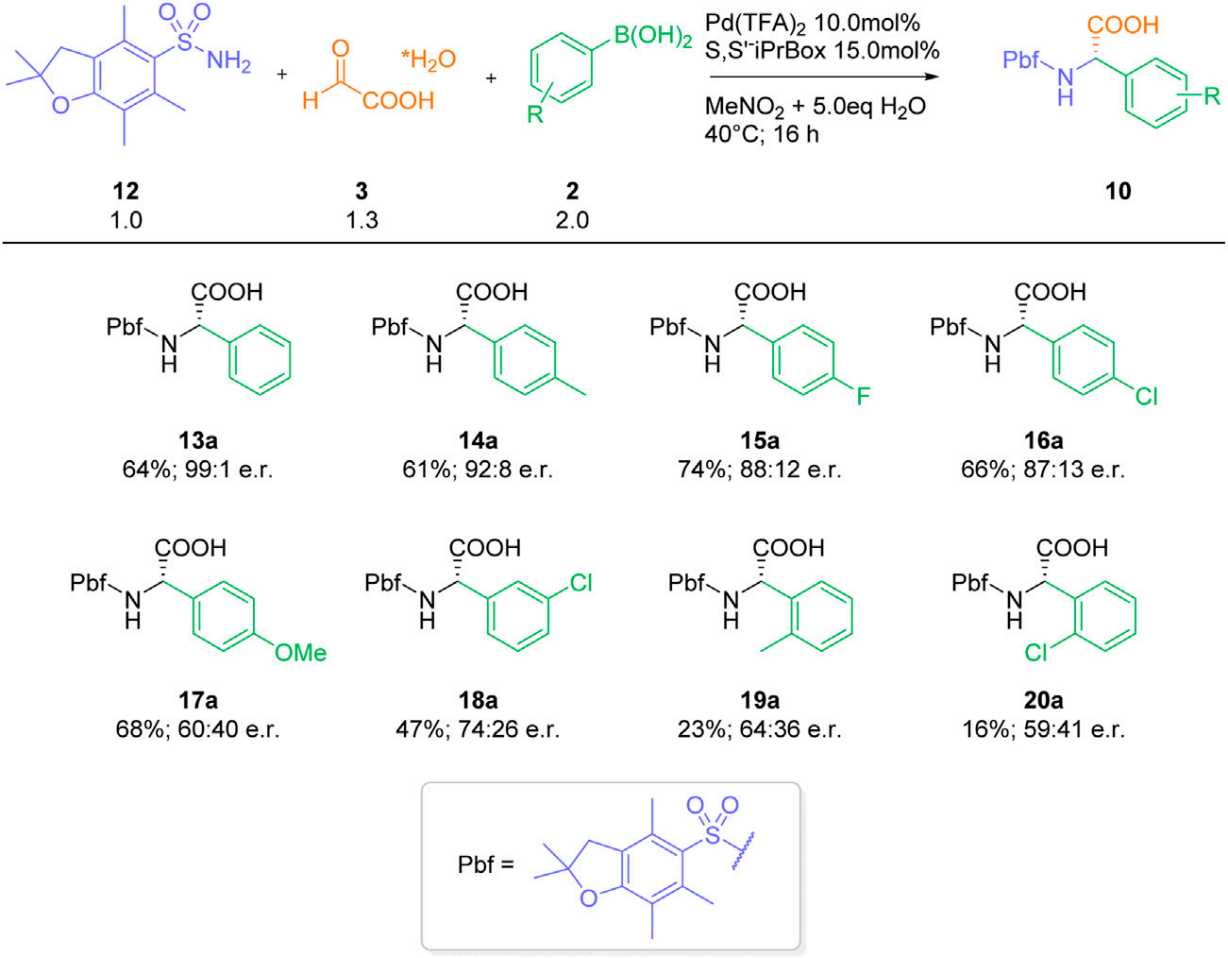
Substrate scope arylboronic acids.

With this in mind, we can provide reasonable explanations for our experimental observations. *Role of solvents*: Whereas the uncatalyzed reaction proceeds well in various different solvents (Diehl et al., 2018), only nitromethane is a suitable solvent for the palladium-catalyzed enantioselective transformation. Nitromethane is known as the solvent of choice for the stabilization of cationic species (Miyaura, 2002; Nishikata, Yamamoto and Miyaura, 2003). We assume, that nitromethane plays a crucial role in stabilizing the active palladium catalyst 31, thereby facilitating the palladium-catalyzed process. We have already observed a similar beneficial effect of nitromethane in other palladium (II)-catalyzed 1, 2-addition reactions (Carpino et al., 1993). *Water as additive*: The product decomplexation step plays a crucial (and often overlooked) role in palladium-catalyzed addition reactions (Holder et al., 2013). Without this final step, no regeneration of the active catalyst will take place. We assume - that water plays a crucial role in the decomplexation step. In the absence of water, the liberation of the active catalyst is somewhat slow, thereby favoring the uncatalyzed racemic background reaction. *Ligands and Palladium Precursors:* As one can derive from Table 3, the formation of a cationic palladium species 31 can be best achieved by using Pd(II) precursors with labile ligands, such as trifluoroacetate. A cationic Pd (II) center with a labile TFA ligand is also ideally suited both for a rapid transmetallation and efficient imine coordination step. The surprising observation that only a single tested ligand (L1) leads to a significant stereoinduction can be rationalized by a matching combination of bite angel and steric bulk. Ligands with a lower bight angle and/or more bulky residues will lead to a sterically more encumbered Pd (II) center. This in turn should lead both to a slower transmetalation step and more difficult coordination of the imine to a Pd(II) complex of type 32. *Influence of the aryl boronic acid:* It is well known, that electron-rich (hetero) aryl boronic acids perform well in traditional, uncatalyzed Petasis reactions (Candejas et al., 2010; Paul, Presset and Le Gall, 2017; Wu, Givskov and Nielsen, 2019). On the other hand, electron-deficient arylboronic acids usually don’t react in the absence of a catalyst or activating agent. This reactivity pattern can be seen in the stereochemical outcome of reactions with different aryl boronic acids (Scheme 3). The electron-rich para-methoxybenzene boronic acid affords *a*-arylgylcine 17a in almost racemic form, presumably due to a fast uncatalyzed addition to the imine 28 *via* pathway A. Reactions with more or less electron-neutral aryl boronic acids 14–16 proceed efficiently *via* pathway B, furnishing the arylglycines 14a-16a in 61%–74% yield and an enantiomeric ratio ≥88:12. On the other hand, an electron-deficient aryl boronic acids 18 furnish the desired product 18a in lower yield and partially decreased enantioselectivity. We assume, that in these cases the transmetallation step is slower, leading to a less efficient catalytic cycle and a comparably fast (or slow) background reaction. For the sterically hindered boronic acids, the corresponding transmetallation step should be even slower, resulting in an even less efficient catalytic cycle. The obtained yields and enantioselectivities for the two arylglycines 19a and 20a align with our rationale. *Lower yields with palladium-catalysis:* Compared to our initial, metal-free reaction (Diehl et al., 2018) we observe significantly reduced yields (typically 20%–40% lower) of the desired arylgylcines in the presence of the palladium-catalyst system. Close analysis of the reaction mixtures revealed the formation of a significant amount of protodeborylated products of type 34. Depending on the reaction up to 80% (based on the amount of used boronic acid) of the deborylated species 34 could be detected by GC and GC/MS analysis. In addition, ^1^H-NMR analysis of the crude reaction mixture showed only traces of boronic acid. In contrast, no extensive deborylation was observed in the absence of a palladium catalyst. Therefore, we assume, that in the presence of a palladium catalyst a third non-productive pathway C, a palladium-catalyzed protodeborylation *via* aryl palladium species 32 is operative. This side reaction leads to a less efficient process in terms of final isolated yields and can result in an unproductive outcome in case both pathways A and B are not favored. From Scheme 6, one can clearly delineate, that achieving an overall efficient and highly enantioselective three-component reaction requires careful adjustment of several, competing factors.

**SCHEME 4:**
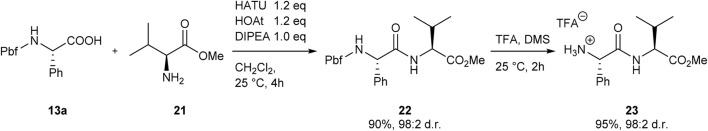
Synthesis of Model dipeptide 23.

**SCHEME 5:**
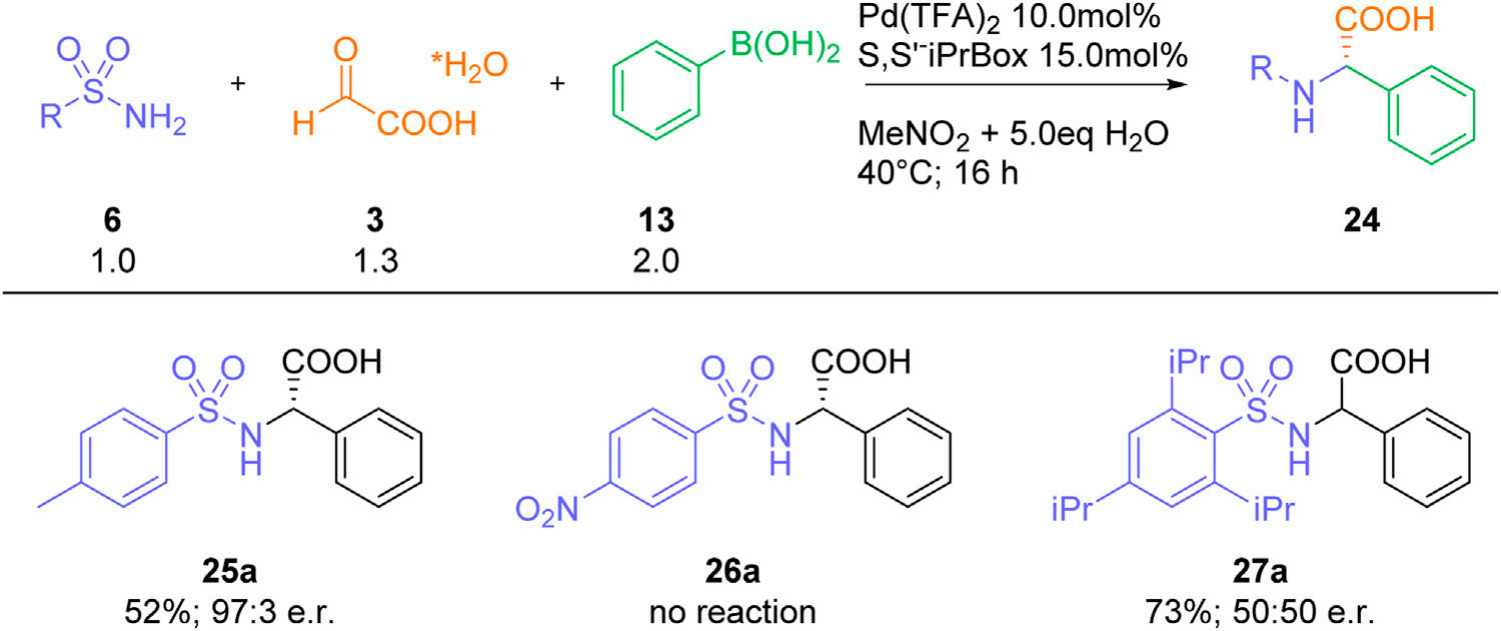
Reactions with selected sulfonamides.

**SCHEME 6:**
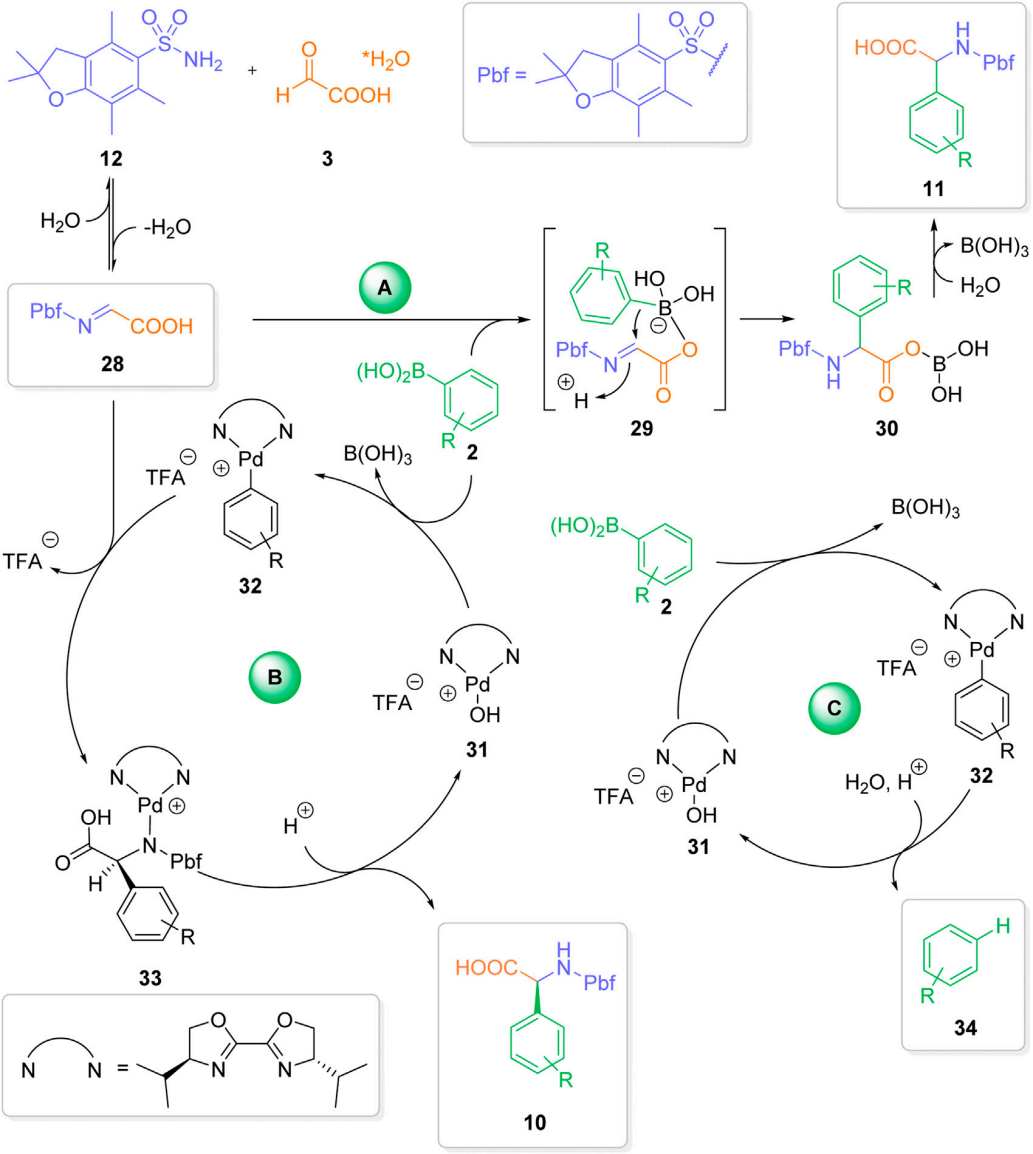
Mechanistic considerations.

Since the three-component reaction proceeds both in the presence and the absence of the palladium catalyst and the enantioselectivity is strongly influenced by various factors (ligand, solvent, type of boronic acid or sulfonamide), one has to consider a more complex mechanistic scenario (Scheme 6).

## 3 Material and methods

### 3.1 Experimental

Unless otherwise mentioned, all reactions were carried out without any precautions to exclude ambient air or moisture. Thin layer chromatography (TLC) was performed on precoated aluminum sheets (TLC silica gel 60 F_254_). The spots were visualized by ultraviolet light, iodine or cerium (IV) ammonium molybdate. Flash column chromatography was performed using a puriflash XS 420 + Flash purifier machine from Interchim with prepacked flash columns (puriFlash_Silica HP_15 µm_F0040, puriFlash PF C18HP 30 µm F0012) and the respectively solvent mixture. All yields refer to the isolated yields of compounds estimated to be >95% pure as determined by ^1^H NMR.

### 3.2 Materials

Unless noted, all starting material were purchased from different commercial sources and used without further purification. Sulfonamide 12 (Carpino et al., 1993) and ligand L1 (Denmark et al., 1997) were synthesized according to known literature procedures. Racemic products for chiral HPLC analysis were prepared according to the same typical procedures reported for the enantioselective 3-component reactions by utilizing the corresponding sulfonamide (0.5 mmol), glyoxylic acid (0.65 mmol) and arylboronic acids (1.0 mmol) in nitromethane (2.0 ml) at 60°C for 24 h.

### 3.3 Absolute configuration

The absolute configuration of the *a*-arylglycines were determined *via* crystal structure of compound 25a. The crystal data are equivalent to the according literature (Duarte-Hernández et al., 2015).

### 3.4 Analytical data and instrumentation

*NMR spectroscopy*—Proton nuclear magnetic resonance spectra (^1^H NMR) and carbon spectra (^13^C NMR) were recorded at a frequency of 400 MHz (^1^H) and 101 MHz (^13^C), respectively. Chemical shifts are expressed as parts of million downfield shift on the *d* scale and are referenced to the solvent peak (Chloroform-d_1_: *d* = 7.26 ppm for ^1^H, *d* = 77.16 ppm for ^13^C; DMSO-d_6_: *d* = 2.50 ppm for ^1^H, *d* = 39.52 ppm for ^13^C). ^19^F NMR spectra were recorded proton decoupled at a frequency of 282 MHz. Chemical shifts are quoted in parts per million and are not referenced. Coupling constants *J*) are quoted in Hz and the observed signal multiplicities are reported as follows: s = singlet, d = doublet, t = triplet, q = quartet, m = multiplet. *Mass spectrometry*—Mass spectra (MS) were measured using ESI (electrospray ionization) techniques. High resolution mass spectra (HRMS) were acquired on a Waters GCT Premium using electron ionization mass spectroscopy (EI-MS-TOF). *Infrared spectroscopy*—Infrared spectra (IR) were recorded on a FT-IR (Fourier transform infrared spectroscopy) spectrometer including a diamond universal ATR sampling technique (attenuated total reflectance) from 4,000–400 cm^−1^. The absorption bands were reported in wave numbers (cm^−1^). *Optical rotations*—Rotation values *α*) were measured using with an analog type *243B polarimeter* from *PerkinElmer*, equipped with a sodium lamp source (589 nm), at 20°C in 10 cm cell and the indicated solvent. The specific rotation values are reported as [α]_λ_^T^ (mass concentration *c*) in g*100 ml^−1^, solvent) and are quoted in deg*mL*dm^−1^*g^−1^. *Analytical chiral HPLC*–Enantiomeric ratios (e. r.) and accordingly enantiomeric excesses (e. e.) were determined by normal phase high performance liquid chromatographic (HPLC) analysis with a *Hewlett Packard*™ system (G1322 A degasser, G1311 quadruple pump, G1316 A diode array detector with visualization at 254 nm) and the use of a Chiralpak^®^ IA, Chiralcel^®^ OD-H or OJ-H as chiral column (4.6 mm × 25 cm) obtained from *Daicel Chemical Industries, Ltd*. Elution conditions for specific compounds are reported in the SI. *Melting points*—Melting points are uncorrected.

### 3.5 General procedures (GP)

GP1 (Initial experiments)—A 10 ml screw cap glass vial was charged with a magnetic stirring bar, sulfonamide 12 (0.50 mmol, 1.0 equiv), glyoxylic acid (0.65 mmol, 1.3 equiv), phenylboronic acid (1.00 mmol, 2.0 equiv), Pd (TFA)_2_ (25 μmol, 0.05 equiv), *S,S′*-*i*PrBox L1 (37.5 µmol, 0.075 equiv) and nitromethane (0.25 M referring to sulfonamide, 2 ml) as solvent. Then the vial was closed with a Teflon lined screw cap and the resulting reaction mixture was stirred at 40°C for 16 h. After cooling to room temperature, the reaction mixture was diluted with acetone and filtered through a short plug of celite and silica gel. The filter pad was rinsed with additional acetone and the combined filtrates were concentrated under reduced pressure. Purification of the crude residue by flash column chromatography afforded the analytically pure product.

GP2 (Ligand variation)—A 10 ml screw cap glass vial was charged with a magnetic stirring bar, sulfonamide 12 (0.50 mmol, 1.0 equiv), glyoxylic acid (0.65 mmol, 1.3 equiv), phenylboronic acid (1.00 mmol, 2.0 equiv), Pd (TFA)_2_ (25 μmol, 0.05 equiv), ligand (37.5 µmol, 0.075 equiv) and nitromethane (0.25 M referring to sulfonamide, 2 ml) as solvent. Then the vial was closed with a Teflon lined screw cap and the resulting reaction mixture was stirred at 40°C for 16 h. After cooling to room temperature, the reaction mixture was diluted with acetone and filtered through a short plug of celite and silica gel. The filter pad was rinsed with additional acetone and the combined filtrates were concentrated under reduced pressure. Purification of the crude residue by flash column chromatography afforded the analytically pure product.

GP3 (Parameter optimization)—A 10 ml screw cap glass vial was charged with a magnetic stirring bar, sulfonamide 12 (0.50 mmol, 1.0 equiv), glyoxylic acid (0.65 mmol, 1.3 equiv), phenylboronic acid (1.00 mmol, 2.0 equiv), Pd (TFA)_2_ (25 μmol, 0.05 equiv), ligand (37.5 µmol, 0.075 equiv) and nitromethane (0.25 M referring to sulfonamide, 2 ml) as solvent. Then the vial was closed with a Teflon lined screw cap and the resulting reaction mixture was stirred at 40°C for 16 h. After cooling to room temperature, the reaction mixture was diluted with acetone and filtered through a short plug of celite and silica gel. The filter pad was rinsed with additional acetone and the combined filtrates were concentrated under reduced pressure. Purification of the crude residue by flash column chromatography afforded the analytically pure product.

GP4 (Boronic acid variation)—A 10 ml screw cap glass vial was charged with a magnetic stirring bar, Pd (TFA)_2_ (50 μmol, 0.1 equiv), L1 (75 μmol, 0.15 equiv) and nitromethane (1 ml) as solvent. After 30 min at 40°C the sulfonamide 12 (0.50 mmol, 1.0 equiv), glyoxylic acid (0.65 mmol, 1.3 equiv), arylboronic acid (1.00 mmol, 2.0 equiv) were added and the inner wall was rinsed with 1 ml nitromethane. Then the vial was closed with a Teflon lined screw cap and the resulting reaction mixture was stirred at 40°C for 16 h. After cooling to room temperature, the reaction mixture was diluted with acetone and filtered through a short plug of celite and silica gel. The filter pad was rinsed with additional acetone and the combined filtrates were concentrated under reduced pressure. Purification of the crude residue by flash column chromatography afforded the analytically pure product.

GP5 (Peptide-coupling)—A 50 ml round flask was charged with a magnetic stirring bar, 13a (1.0 equiv, 0.48 mmol, 200.0 mg), HOAt (1.2 equiv, 0.58 mmol, 78.0 mg), HATU (1.2 equiv, 0.58 mmol, 222.0 mg) and dichlormethane (5 ml) and stirred at room temperature for 10 min. A mixture of l-valine methyl ester hydrochloride (1.0 eq, 0.48 mmol, 96.0 mg) and DIPEA (1.1 equiv, 0.48 mmol, 0.1 ml) in dichlormethane (5 ml) were added and the reaction mixture was stirred for 4 h at room temperature. The resulting reaction mixture was washed with saturated NaCl solution (2 × 10 ml). The organic phase was dried over Na_2_SO_4_ and concentrated under reduced pressure. Purification of the crude residue by flash column chromatography afforded the analytically pure product.

GP6 (Deprotecting of Pbf-group)—The *N*-Pbf-group was removed by a method according to known literature.^1^ The *N-*Pbf-protected *a*-arylglycine-derivative (1.0 eq) was added to a suitable round flask and a solution of TFA (69 equiv, 23.4 mmol, 1.8 ml) and DMS (8.0 equiv, 2.7 mmol, 0.2 ml).

GP7 (Sulfonamide variation)—A 10 ml screw cap glass vial was charged with a magnetic stirring bar, Pd (TFA)_2_ (16.6 mg, 50 μmol, 0.1 equiv), L1 (16.8 mg, 75 μmol, 0.15 equiv) and nitromethane (1 ml) as solvent. After 30 min at 40°C the sulfonamide (0.50 mmol, 1.0 equiv), glyoxylic acid (59.8 mg, 0.65 mmol, 1.3 equiv), phenylboronic acid (121.9 mg, 1.00 mmol, 2.0 equiv) were added and the inner wall was rinsed with 1 ml nitromethane. Then the vial was closed with a Teflon lined screw cap and the resulting reaction mixture was stirred at 40°C for 16 h. After cooling to room temperature, the reaction mixture was diluted with acetone and filtered through a short plug of celite and silica gel. The filter pad was rinsed with additional acetone and the combined filtrates were concentrated under reduced pressure. Purification of the crude residue by flash column chromatography afforded the analytically pure product.

## 4 Conclusion

In conclusion, we have described a novel, palladium-catalyzed enantioselective three-component reaction of aryl boronic acids, sulfonamides and glyoxylic acid. This method provides access to *a*-arylglycines in moderate to good yields and enantioselectivities. The formed Pbf-protected products can be directly utilized as building blocks for the synthesis of arylglycine-containing peptides. Although the scope of this process is still limited, our tailored catalyst system allows an enantioselective preparation of different *a*-arylglycines despite a fast racemic background reaction. Detailed mechanistic considerations reveal a complex mechanistic scenario with several competing pathways. Currently, a more thorough study of the reaction mechanism(s) is underway in our laboratory. The more detailed mechanistic insight will be used to develop a more general version of the herein-reported three-component reaction. Jiang and Schaus, 2017.

## Data Availability

The original contributions presented in the study are included in the article/Supplementary Material, further inquiries can be directed to the corresponding author.
