# Supplementary material for: Palladium-catalyzed asymmetric three-component reaction between glyoxylic acid, sulfonamides and arylboronic acids for the synthesis of α-arylglycine derivatives
Source: Front Chem. 2023 Mar 13;11:1165618. doi: 10.3389/fchem.2023.1165618 (PMC10040839; doi:10.3389/fchem.2023.1165618)
Supplement: Supplementary file 1 [file DataSheet1.pdf]

Supplementary Material

Palladium-Catalyzed Asymmetric Three-Component Reaction between Glyoxylic Acid, Sulfonamides and Arylboronic Acids for the Synthesis of α-Arylglycine Derivatives

Bastian Jakob, Andreas M. Diehl, Kathrin Horst, Harald Kelm, Georg Manolikakes*

*** Correspondence:** Corresponding Author: manolikakes@chemie.uni-kl.de

1 General Information

Experimental

Unless otherwise mentioned, all reactions were carried out without any precautions to exclude ambient air or moisture. Thin layer chromatography (TLC) was performed on precoated aluminum sheets (TLC silica gel 60 F_{254}). The spots were visualized by ultraviolet light, iodine or cerium(IV) ammonium molybdate. Flash column chromatography was performed using a puriflash XS 420+ Flash purifier machine from Interchim with prepacked flash columns (Puriflash_Silica HP_15 µm_F0040, Puriflash PF C18HP 30 µm F0012) and the respectively solvent mixture. All yields refer to the isolated yields of compounds estimated to be > 95% pure as determined by ^{1}H NMR.

Materials

Unless noted, all starting material were purchased from different commercial sources and used without further purification. Sulfonamide **12** and ligand **L1** were synthesized according to known literature procedures.[1,2] Racemic products for chiral HPLC analysis were prepared according to the same typical procedures reported for the enantioselective 3-component reactions by utilizing the corresponding sulfonamide (0.5 mmol), glyoxylic acid (0.65 mmol) and arylboronic acids (1.0 mmol) in nitromethane (2.0 mL) at 60 °C for 24h.

Absolute configuration

The absolute configuration of the α-arylglycines were determined via crystal structure of compound **25a**. The crystal data are equivalent to known literature.[3]

Analytical Data and Instrumentation

NMR spectroscopy - Proton nuclear magnetic resonance spectra (^{1}H NMR) and carbon spectra (^{13}C NMR) were recorded at a frequency of 400 MHz (^{1}H) and 101 MHz (^{13}C), respectively. Chemical shifts are expressed as parts of million downfield shift on the δ scale and are referenced to the solvent peak (Chloroform-d_1: δ = 7.26 ppm for ^{1}H, δ = 77.16 ppm for ^{13}C; DMSO-d_6: δ = 2.50 ppm for ^{1}H, δ = 39.52 ppm for ^{13}C). ^{19}F NMR spectra were recorded proton decoupled at a frequency of 282 MHz. Chemical shifts are quoted in parts per million and are not referenced. Coupling constants (*J*) are quoted

in Hz and the observed signal multiplicities are reported as follows: s = singlet, d = doublet, t = triplet, q = quartet, m = multiplet.

Mass spectrometry - Mass spectra (MS) were measured using ESI (electrospray ionization) techniques. High resolution mass spectra (HRMS) were acquired on a Waters GCT Premium using electron ionization mass spectroscopy (EI-MS-TOF).

Infrared spectroscopy - Infrared spectra (IR) were recorded on a FT-IR (Fourier transform infrared spectroscopy) spectrometer including a diamond universal ATR sampling technique (attenuated total reflectance) from 4000-400 cm^{-1}. The absorption bands were reported in wave numbers (cm^{-1}).

Optical rotations - Rotation values (α) were measured using with an analog type *243B polarimeter* from *PerkinElmer*, equipped with a sodium lamp source (589 nm), at 20 °C in 10 cm cell and the indicated solvent. The specific rotation values are reported as $[\alpha]_\lambda^T$ (mass concentration (*c*) in g*100 mL^{-1}, solvent) and are quoted in deg*mL*dm^{-1}*g^{-1}.

Analytical chiral HPLC – Enantiomeric ratios (e.r.) and accordingly enantiomeric excesses (e.e.) were determined by normal phase high performance liquid chromatographic (HPLC) analysis with a *Hewlett Packard*™ system (G1322A degasser, G1311 quadruple pump, G1316A diode array detector with visualization at 254 nm) and the use of a Chiralpak® IA, Chiralcel® OD-H or OJ-H as chiral column (4.6 mm x 25 cm) obtained from *Daicel Chemical Industries, Ltd*. Elution conditions for specific compounds are reported in the SI.

Melting points - Melting points are uncorrected.

2 Preparation and analytical data

2.1 General procedures (GP)

GP1 (Initial experiments) – A 10 mL screw cap glass vial was charged with a magnetic stirring bar, sulfonamide **12** (134.7 mg, 0.50 mmol, 1.0 equiv), glyoxylic acid (59.8 mg, 0.65 mmol, 1.3 equiv), phenylboronic acid (121.9 mg, 1.00 mmol, 2.0 equiv), $Pd(TFA)_2$ (8.3 mg, 25 µmol, 0.05 equiv), S,S'-*i*PrBox **L1** (8.4 mg, 37.5 µmol, 0.075 equiv) and nitromethane (0.25 M referring to sulfonamide, 2 mL) as solvent. Then the vial was closed with a teflon lined screw cap and the resulting reaction mixture was stirred at 40 °C for 16 h. After cooling to room temperature, the reaction mixture was diluted with acetone and filtered through a short plug of celite and silica gel. The filter pad was rinsed with additional acetone and the combined filtrates were concentrated under reduced pressure. Purification of the crude residue by flash column chromatography afforded the analytically pure product.

GP2 (Ligand variation) – A 10 mL screw cap glass vial was charged with a magnetic stirring bar, sulfonamide **12** (134.7 mg, 0.50 mmol, 1.0 equiv), glyoxylic acid (59.8 mg, 0.65 mmol, 1.3 equiv), phenylboronic acid (121.9 mg, 1.00 mmol, 2.0 equiv), $Pd(TFA)_2$ (8.3 mg, 25 µmol, 0.05 equiv), ligand (37.5 µmol, 0.075 equiv) and nitromethane (0.25 M referring to sulfonamide, 2 mL) as solvent. Then the vial was closed with a teflon lined screw cap and the resulting reaction mixture was stirred at 40 °C for 16 h. After cooling to room temperature, the reaction mixture was diluted with acetone and filtered through a short plug of celite and silica gel. The filter pad was rinsed with additional acetone and the combined filtrates were concentrated under reduced pressure. Purification of the crude residue by flash column chromatography afforded the analytically pure product.

GP3 (Parameter optimization) – A 10 mL screw cap glass vial was charged with a magnetic stirring bar, sulfonamide **12** (134.7 mg, 0.50 mmol, 1.0 equiv), glyoxylic acid (59.8 mg, 0.65 mmol, 1.3 equiv), phenylboronic acid (121.9 mg, 1.00 mmol, 2.0 equiv), $Pd(TFA)_2$ (8.3 mg, 25 µmol, 0.05 equiv), **L1** (8.4 mg, 37.5 µmol, 0.075 equiv) and nitromethane (0.25 M referring to sulfonamide, 2 mL) as solvent. Then the vial was closed with a teflon lined screw cap and the resulting reaction mixture was stirred at 40 °C for 16 h. After cooling to room temperature, the reaction mixture was diluted with acetone and filtered through a short plug of celite and silica gel. The filter pad was rinsed with additional acetone and the combined filtrates were concentrated under reduced pressure. Purification of the crude residue by flash column chromatography afforded the analytically pure product.

GP4 (Boronic acid variation) – A 10 mL screw cap glass vial was charged with a magnetic stirring bar, $Pd(TFA)_2$ (16.6 mg, 50 µmol, 0.1 equiv), **L1** (16.8 mg, 75 µmol, 0.15 equiv) and nitromethane (1 mL) as solvent. After 30 min at 40 °C the sulfonamide **12** (134.7 mg, 0.50 mmol, 1.0 equiv), glyoxylic acid (59.8 mg, 0.65 mmol, 1.3 equiv), arylboronic acid (1.00 mmol, 2.0 equiv) were added and the inner wall was rinsed with 1 mL nitromethane. Then the vial was closed with a teflon lined screw cap and the resulting reaction mixture was stirred at 40 °C for 16 h. After cooling to room temperature, the reaction mixture was diluted with acetone and filtered through a short plug of celite and silica gel. The filter pad was rinsed with additional acetone and the combined filtrates were concentrated under reduced pressure. Purification of the crude residue by flash column chromatography afforded the analytically pure product.

GP5 (Peptide-coupling) – A 50 mL round flask was charged with a magnetic stirring bar, **13a** (1.0 equiv, 0.48 mmol, 200.0 mg), HOAt (1.2 equiv, 0.58 mmol, 78.0 mg), HATU (1.2 equiv, 0.58 mmol, 222.0 mg) and dichlormethane (5 mL) and stirred at room temperature for 10 min. A mixture of L-valine methyl ester hydrochloride (1.0 eq, 0.48 mmol, 96.0 mg) and DIPEA (1.1 equiv, 0.48 mmol, 0.1 mL) in dichlormethane (5 mL) were added and the reaction mixture was stirred for 4 hours at room temperature. The resulting reaction mixture was washed with saturated NaCl solution (2 x 10 mL). the organic phase was dried over Na_2SO_4 and concentrated under reduced pressure. Purification of the crude residue by flash column chromatography afforded the analytically pure product.

GP6 (Deprotecting of Pbf-group) – The *N*-Pbf-group was removed by a method according to known literature.[1] The *N*-Pbf-protected α-arylglycine-derivative (1.0 eq) was added to a suitable round flask and a solution of TFA (69 equiv, 23.4 mmol, 1.8 mL) and DMS (8.0 equiv, 2.7 mmol, 0.2 mL)

GP7 (Sulfonamide variation) – A 10 mL screw cap glass vial was charged with a magnetic stirring bar, $Pd(TFA)_2$ (16.6 mg, 50 µmol, 0.1 equiv), **L1** (16.8 mg, 75 µmol, 0.15 equiv) and nitromethane (1 mL) as solvent. After 30 min at 40 °C the sulfonamide (0.50 mmol, 1.0 equiv), glyoxylic acid (59.8 mg, 0.65 mmol, 1.3 equiv), phenylboronic acid (121.9 mg, 1.00 mmol, 2.0 equiv) were added and the inner wall was rinsed with 1 mL nitromethane. Then the vial was closed with a teflon lined screw cap and the resulting reaction mixture was stirred at 40 °C for 16 h. After cooling to room temperature, the reaction mixture was diluted with acetone and filtered through a short plug of celite and silica gel. The filter pad was rinsed with additional acetone and the combined filtrates were concentrated under reduced pressure. Purification of the crude residue by flash column chromatography afforded the analytically pure product.

2.2 α-Arylglycines

2.2.1

(S)-2-((2,2,4,6,7-pentamethyl-2,3-dihydrobenzofuran)-5-sulfonamido)-2-phenylacetic acid **13a**

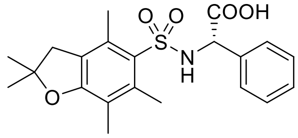

13a

Prepared according to **GP4** from phenylboronic acid (2.0 equiv, 1.00 mmol, 121.9 mg). Purification by reverse phase column chromatography (H_2O/MeCN + 0.1 vol% TFA = 9:1 → 2:8) and freeze drying afterwards afforded product **13a** as a colorless solid (130 mg, 64%).

$[\alpha]_D^{20}$ = +91.84 (c 0.1, $CHCl_3$)

e.r = 99:1 [HPLC conditions: Chiralcel ® IA column, *n*-hexane/ethanol/TFA = 9:1:0.1, flow rate = 0.7 mL/min, t_R (minor) = 19.3 min and t_R (major) = 20.8 min].

R_f (*n*-hexane/acetone/AcOH = 2:1:0.1) 0.31

m.p. 78-80 °C

^{1}H NMR (400 MHz, $CDCl_3$) δ = 7.23 – 7.13 (m, 5H), 5.75 (s, 1H), 4.98 (d, 1H, J = 4 Hz), 2.87 (s, 2H), 2.45 (s, 3H), 2.37 (s, 3H), 2.01 (s, 3H), 1.45 (s, 6H).

^{13}C NMR (101 MHz, $CDCl_3$) δ = 174.92, 159.97, 139.64, 134.93, 134.25, 128.72, 128.64, 127.78, 127.27, 125.16, 118.11, 86.98, 59.13, 43.15, 28.66, 28.64, 19.39, 17.77, 12.50.

MS (APCI) m/z calcd for $C_{21}H_{25}NO_5S$ 404.5 $[M+H]^+$, found 404.3 $[M+H]^+$

HRMS (TOF MS EI+) m/z calcd for $C_{21}H_{25}NO_5S$ 403.1453 $[M^+]$, found 403.1455 $[M^+]$

IR (ν in cm^{-1}): 3266, 2970, 2927, 1734, 1575, 1455, 1409, 1371, 1239, 1208, 1158, 1138, 1089, 993, 850, 782, 733, 698, 639, 522.

2.2.2

(S)-2-((2,2,4,6,7-pentamethyl-2,3-dihydrobenzofuran)-5-sulfonamido)-2-(p-tolyl)acetic acid **14a**

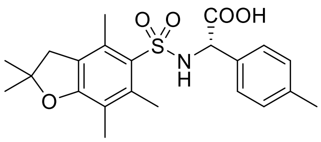

14a

Prepared according to **GP4** from p-tolylboronic acid (136.0 mg, 1.00 mmol, 2.0 equiv). Purification by reverse phase column chromatography (H_2O/MeCN + 0.1 vol% TFA = 9:1 → 2:8) and freeze drying afterwards afforded product **14a** as a colorless solid (128 mg, 61%).

$[\alpha]_D^{20}$ = +101.0 (c 0.1, $CHCl_3$)

e.r = 92:8 [HPLC conditions: Chiralcel ® IA column, *n*-hexane/ethanol/TFA = 9:1:0.1, flow rate = 0.7 mL/min, t_R (minor) = 20.7 min and t_R (major) = 22.7 min].

R_f (*n*-hexane/acetone/AcOH = 2:1:0.1) 0.36

m.p. 160-163 °C

^{1}H NMR (400 MHz, $CDCl_3$) δ = 7.26 – 6.99 (m, 5H), 5.66 (s, 1H), 4.93 (d, *J* = 4 Hz, 1H), 2.89 (s, 2H), 2.44 (s, 3H), 2.39 (s, 3H), 2.27 (s, 3H) 2.01 (s, 3H), 1.45 (d, *J* = 4 Hz, 6H).

^{13}C NMR (101 MHz, $CDCl_3$) δ = 175.15, 159.94, 139.63, 138.72, 134.24, 131.97, 129.34, 127.89, 127.14, 125.15, 118.08, 86.94, 58.88, 43.19, 28.61, 21.24, 19.41, 17.78, 12.47.

MS (APCI) m/z calcd for $C_{22}H_{27}NO_5S$ 418.2 $[M+H]^+$, found 418.3 $[M+H]^+$

HRMS (TOF MS EI+) m/z calcd for $C_{22}H_{27}NO_5S$ 417.1610 $[M^+]$, found 417.1612 $[M^+]$

IR (ν in cm^{-1}): 3266, 2970, 2927, 1734, 1575, 1455, 1409, 1371, 1239, 1208, 1158, 1138, 1089, 993, 850, 782, 733, 698, 639, 522.

2.2.3

(S)-2-(4-fluorophenyl)-2-((2,2,4,6,7-pentamethyl-2,3-dihydrobenzofuran)-5-sulfonamido)acetic acid **15a**

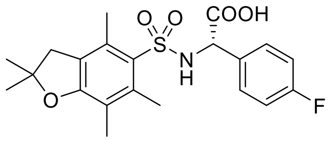

15a

Prepared according to **GP4** from (4-fluorophenyl)boronic acid (139.9 mg, 1.00 mmol, 2.0 equiv). Purification by reverse phase column chromatography (H_2O/MeCN + 0.1 vol% TFA = 9:1 → 2:8) and freeze drying afterwards afforded product **15a** as a colorless solid (155 mg, 74%).

$[\alpha]_D^{20}$ = +78.3 (c 0.1, $CHCl_3$)

e.r = 88:12 [HPLC conditions: Chiralcel ® IA column, *n*-hexane/ethanol/TFA = 9:1:0.1, flow rate = 0.7 mL/min, t_R (minor) = 18.9 min and t_R (major) = 22.6 min].

R_f (*n*-hexane/acetone/AcOH = 2:1:0.1) 0.34

m.p. 150-152 °C

^{1}H NMR (400 MHz, $CDCl_3$) δ = 7.15 – 7.11 (m, 2H), 6.90-6.88 (m, 2H) 5.71 (s, 1H), 5.00 (d, 1H, *J* = 4 Hz), 2.88 (s, 2H), 2.47 (s, 3H), 2.35 (s, 3H), 2.03 (s, 3H), 1.45 (s, 6H).

^{13}C NMR (101 MHz, $CDCl_3$) δ = 174.03, 163.97, 161.50, 159.94, 139.44, 134.09, 130.68, 130.65, 129.08, 128.99, 127.66, 125.08, 118.07, 115.56, 115.34, 86.94, 58.30, 43.02, 28.51, 28.46, 19.25, 17.66, 12.38.

^{19}F NMR (376 MHz, $CDCl_3$) δ = -112.59

MS (APCI) m/z calcd for $C_{21}H_{24}FNO_5S$ 434.2 $[M+H]^+$, found 434.3 $[M+H]^+$

HRMS (TOF MS EI+) m/z calcd for $C_{21}H_{24}FNO_5S$ 403.1453 $[M^+]$, found 403.1455 $[M^+]$

IR (v in cm^{-1}): 2970, 1737, 1575, 1447, 1369, 1218, 1135, 1089, 850, 780, 732, 660, 639, 543.

2.2.4

(S)-2-(4-chlorophenyl)-2-((2,2,4,6,7-pentamethyl-2,3-dihydrobenzofuran)-5-sulfonamido)acetic acid **16a**

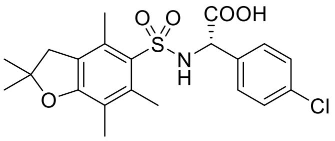

16a

Prepared according to **GP4** from (4-chlorophenyl)boronic acid (156.4 mg, 1.00 mmol, 2.0 equiv). Purification by reverse phase column chromatography (H_2O/MeCN + 0.1 vol% TFA = 9:1 → 2:8) and freeze drying afterwards afforded product **16a** as a colorless solid (145 mg, 66%).

$[\alpha]_D^{20}$ = +81.7 (c 0.1, $CHCl_3$)

e.r = 87:13 [HPLC conditions: Chiralcel ® IA column, *n*-hexane/ethanol/TFA = 9:1:0.1, flow rate = 0.7 mL/min, t_R (minor) = 19.4 min and t_R (major) = 23.8 min].

R_f (*n*-hexane/acetone/AcOH = 2:1:0.1) 0.36

m.p. 165-168 °C

^{1}H NMR (400 MHz, $CDCl_3$) δ = 7.16 (d, *J* = 8 Hz, 2H), 7.09 (d, *J* = 8 Hz, 2H), 5.74 (d, *J* = 8 Hz, 1H), 4.99 (d, *J* = 8 Hz, 1H), 2.88 (s, 2H), 2.46 (s, 3H), 2.34 (s, 3H), 2.03 (s, 3H), 1.46 (d, *J* = 8 Hz, 6H).

^{13}C NMR (101 MHz, $CDCl_3$) δ = 173.74, 160.11, 139.56, 134.79, 133.48, 128.74, 127.76, 125.24, 118.24, 87.11, 58.51, 43.15, 28.65, 28.61, 19.39, 17.81, 12.51.

MS (APCI) m/z calcd for $C_{21}H_{24}ClNO_5S$ 438.1 $[M+H]^+$, found 438.3 $[M+H]^+$

HRMS (TOF MS EI+) m/z calcd for $C_{21}H_{24}ClNO_5S$ [^{35}Cl] 437.1064 [M^+], found 437.1062 [M^+]; [^{37}Cl] 439.1034 [M^+], found 439.1062 [M^+]

IR (ν in cm^{-1}): 3375, 2970, 2929, 1728, 1694, 1577, 1455, 1368, 1142, 1091, 989, 888, 850, 782, 636, 617, 562, 537.

2.2.5

(S)-2-(4-methoxyphenyl)-2-((2,2,4,6,7-pentamethyl-2,3-dihydrobenzofuran)-5-sulfonamido)acetic acid **17a**

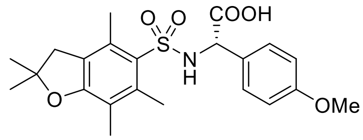

17a

Prepared according to **GP4** from (4-methoxyphenyl)boronic acid (152.0 mg, 1.00 mmol, 2.0 equiv). Purification by reverse phase column chromatography (H_2O/MeCN + 0.1 vol% TFA = 9:1 → 2:8) and freeze drying afterwards afforded product **17a** as a colorless solid (148 mg, 68%).

$[\alpha]_D^{20}$ = +21.7 (c 0.1, $CHCl_3$)

e.r = 60:40 [HPLC conditions: Chiralcel ® IA column, *n*-hexane/ethanol/TFA = 9:1:0.1, flow rate = 0.7 mL/min, t_R (minor) = 24.7 min and t_R (major) = 29.0 min].

R_f (*n*-hexane/acetone/AcOH = 2:1:0.1) 0.31

m.p. 158-161 °C

^{1}H NMR (400 MHz, $CDCl_3$) δ = 7.05 (d, *J* = 8 Hz, 2H), 6.71 (d, *J* = 8 Hz, 2H), 5.62 (d, *J* = 4 Hz, 1H), 4.93 (d, 1H, *J* = 8 Hz), 3.74 (s, 3H), 2.88 (s, 2H), 2.45 (s, 3H), 2.37 (s, 3H), 2.01 (s, 3H), 1.45 (s, 6H).

^{13}C NMR (101 MHz, $CDCl_3$) δ = 174.78, 159.91, 139.56, 134.20, 128.52, 128.03, 126.94, 125.15, 118.08, 114.00, 58.59, 55.37, 43.19, 28.63, 28.57, 19.42, 17.80, 12.50.

MS (APCI) m/z calcd for $C_{22}H_{27}NO_6S$ 434.2 $[M+H]^+$, found 434.3 $[M+H]^+$

HRMS (TOF MS EI+) m/z calcd for $C_{22}H_{27}NO_6S$ 433.1559 $[M^+]$, found 433.1571 $[M^+]$

IR (ν in cm^{-1}): 2970, 1738, 1575, 1511, 1371, 1304, 1231, 1218, 1136, 1088, 780, 732, 638, 615, 529.

2.2.6

(S)-2-(3-chlorophenyl)-2-((2,2,4,6,7-pentamethyl-2,3-dihydrobenzofuran)-5-sulfonamido)acetic acid **18a**

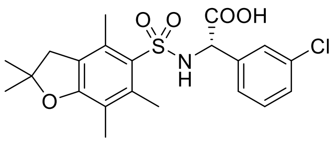

18a

Prepared according to **GP4** from (3-chlorphenyl)boronic acid (156.4 mg, 1.00 mmol, 2.0 equiv). Purification by reverse phase column chromatography (H_2O/MeCN + 0.1 vol% TFA = 9:1 → 2:8) and freeze drying afterwards afforded product **18a** as a colorless solid (103 mg, 47%).

$[\alpha]_D^{20}$ = +47.9 (c 0.1, $CHCl_3$)

e.r = 74:26 [HPLC conditions: Chiralcel ® IA column, *n*-hexane/ethanol/TFA = 9:1:0.1, flow rate = 0.7 mL/min, t_R (minor) = 18.3 min and t_R (major) = 20.5 min].

R_f (*n*-hexane/acetone/AcOH = 2:1:0.1) 0.42

m.p. 123-126 °C

^{1}H NMR (400 MHz, $CDCl_3$) δ = 7.18 – 7.05 (m, 4H), 5.75 (d, *J* = 8 Hz, 1H), 5.00 (d, 1H, *J* = 4 Hz), 2.87 (d, *J* = 8 Hz, 2H), 2.45 (s, 3H), 2.37 (s, 3H), 2.01 (s, 3H), 1.45 (s, 6H).

^{13}C NMR (101 MHz, $CDCl_3$) δ = 174.92, 159.97, 139.64, 134.93, 134.25, 128.72, 128.64, 127.78, 127.27, 125.16, 118.11, 86.98, 59.13, 43.15, 28.66, 28.64, 19.39, 17.77, 12.50.

MS (APCI) m/z calcd for $C_{21}H_{24}ClNO_5S$ 438.1 $[M+H]^+$, found 438.3 $[M+H]^+$

HRMS (TOF MS EI+) m/z calcd for $C_{21}H_{24}ClNO_5S$ [^{35}Cl] 437.1064 [M^+], found 437.1056 [M^+]; [^{37}Cl] 439.1034 [M^+], found 439.1042 [M^+]

IR (v in cm^{-1}): 2970, 2930, 1735, 1575, 1435, 1371, 1286, 1136, 1089, 892, 850, 699, 640, 559.

2.2.7

(S)-2-((2,2,4,6,7-pentamethyl-2,3-dihydrobenzofuran)-5-sulfonamido)-2-(o-tolyl)acetic acid **19a**

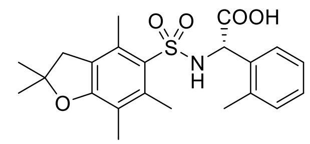

19a

Prepared according to **GP4** from (2-methylphenyl)boronic acid (136.0 mg, 1.00 mmol, 2.0 equiv). Purification by reverse phase column chromatography (H_2O/MeCN + 0.1 vol% TFA = 9:1 → 2:8) and freeze drying afterwards afforded product **19a** as a colorless solid (48 mg, 23%).

$[\alpha]_D^{20}$ = +21.9 (c 0.1, $CHCl_3$)

e.r = 64:36 [HPLC conditions: Chiralcel ® IA column, *n*-hexane/ethanol/TFA = 9:1:0.1, flow rate = 0.7 mL/min, t_R (minor) = 20.3 min and t_R (major) = 23.0 min].

R_f (*n*-hexane/acetone/AcOH = 2:1:0.1) 0.29

m.p. 165-168 °C

^{1}H NMR (400 MHz, $CDCl_3$) δ = 7.15– 6.99 (m, 4H), 5.61 (s, 1H), 5.24 (s, 1H), 2.87 (s, 2H), 2.45 (s, 3H), 2.36 (s, 3H), 2.26 (s, 3H), 2.01 (s, 3H), 1.45 (d, *J* = 2 Hz, 6H).

^{13}C NMR (101 MHz, $CDCl_3$) δ = 174.43, 159.80, 139.56, 136.38, 134.10, 133.33, 130.75, 128.58, 127.73, 126.88, 126.14, 125.02, 117.98, 86.84, 55.51, 43.02, 28.49, .

MS (APCI) m/z calcd for $C_{22}H_{27}NO_5S$ 418.2 $[M+H]^+$, found 418.3 $[M+H]^+$

HRMS (TOF MS EI+) m/z calcd for $C_{22}H_{27}NO_5S$ 417.1610 $[M^+]$, found 417.1614 $[M^+]$

IR (ν in cm^{-1}): 3260, 2970, 1737, 1725, 1575, 1457, 1371, 1294, 1216, 1141, 1088, 902, 850, 639, 555.

2.2.8

(S)-2-(2-chlorophenyl)-2-((2,2,4,6,7-pentamethyl-2,3-dihydrobenzofuran)-5-sulfonamido)acetic acid **20a**

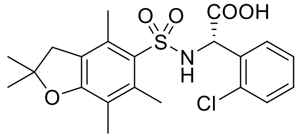

20a

Prepared according to **GP4** from (2-chlorphenyl)boronic acid (156.4 mg, 1.00 mmol, 2.0 equiv). Purification by reverse phase column chromatography (H_2O/MeCN + 0.1 vol% TFA = 9:1 → 2:8) and freeze drying afterwards afforded product **20a** as a colorless solid (35 mg, 16%).

$[\alpha]_D^{20}$ = +33.0 (c 0.1, $CHCl_3$)

e.r = 59:41 [HPLC conditions: Chiralcel ® IA column, *n*-hexane/ethanol/TFA = 9:1:0.1, flow rate = 0.7 mL/min, t_R (minor) = 22.6 min and t_R (major) = 27.3 min].

R_f (*n*-hexane/acetone/AcOH = 2:1:0.1) 0.36

m.p. 123-125 °C

^{1}H NMR (400 MHz, $CDCl_3$) δ = 7.24 – 7.07 (m, 4H), 5.88 (d, *J* = 8 Hz, 1H), 5.38 (d, *J* = 8 Hz, 1H), 2.87 (s, 2H), 2.44 (s, 3H), 2.40 (s, 3H), 1.96 (s, 3H), 1.45 (s, 6H).

^{13}C NMR (101 MHz, $CDCl_3$) δ = 173.19, 159.81, 139.67, 134.36, 133.39, 133.33, 129.71, 127.30, 126.78, 125.00, 117.93, 86.82, 56.59, 43.03, 28.56, 28.54, 19.29, 17.64, 12.32.

MS (APCI) m/z calcd for $C_{21}H_{24}ClNO_5S$ 438.1 $[M+H]^+$, found 438.3 $[M+H]^+$

HRMS (TOF MS EI+) m/z calcd for $C_{21}H_{24}ClNO_5S$ [^{35}Cl] 437.1064 [M^+], found 437.1054 [M^+]; [^{37}Cl] 439.1034 [M^+], found 439.1041 [M^+]

IR (ν in cm^{-1}): 2970, 1737, 1575, 1457, 1369, 1216, 1089, 898, 849, 749, 640, 613, 530.

2.2.9 methyl ((S)-2-((2,2,4,6,7-pentamethyl-2,3-dihydrobenzofuran)-5-sulfonamido)-2-phenylacetyl)-L-valinate **22**

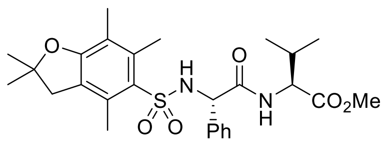

22

Prepared according to **GP5.** Purification by flash column chromatography (*n*-hexane/EtOAc 9:1) afforded product **22** as a colorless solid (223 mg, 90%).

[α]$_D^{20}$ = +59.8 (c 0.9, $CHCl_3$)

d.r = > 98:2; determined via ^{1}H-NMR

R$_f$ (*n*-hexane/EtOAc = 7:3) 0.36

m.p. 106-107 °C

^{1}H NMR (500 MHz, $CDCl_3$) δ = 7.24 – 7.05 (m, 5H), 6.24 (d, *J* = 8.4 Hz, 1H), 5.82 (d, *J* = 4.7 Hz, 1H), 4.75 (d, *J* = 4.8 Hz, 1H), 4.41 (dd, *J* = 8.5 Hz, 1H), 3.64 (s, 3H), 2.88 (s, 2H), 2.44 (s, 3H), 2.37 (s, 3H), 2.14-2.04 (m, 1H), 2.01 (s, 3H), 1.45 (s, 6H), 0.88-0.77 (m, 6H).

^{13}C NMR (126 MHz, $CDCl_3$) δ = 171.6, 169.4, 159.8, 139.5, 136.4, 134.2, 128.8, 128.7, 128.0, 127.6, 125.1, 118.1, 86.9, 60.7, 57.8, 52.3, 43.2, 31.3, 28.7, 28.69, 19.4, 18.9, 17.8, 17.7, 12.5.

MS (ESI) m/z calcd for $C_{27}H_{36}N_2O_7S$ 516.23 [M-H]$^-$, found 515.35 [M-H]$^-$

HRMS (MALDI) m/z calcd for $C_{27}H_{36}N_2O_7S$ 555.19257 [M+K]$^+$, found 555.19022 [M+K]$^+$

IR (v in cm^{-1}): 3288, 2963, 1733, 1645, 1435, 1372, 1318, 1218, 1141, 1082, 988, 782, 733, 694, 661, 640, 612, 556, 521, 507, 467.

2.2.10 (S)-2-(((S)-1-methoxy-3-methyl-1-oxobutan-2-yl)amino)-2-oxo-1-phenylethan-1-aminium trifluoroacetate **23**

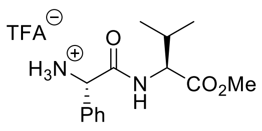

23

Prepared according to **GP6** with **23** (1.0 equiv, 0.34 mmol, 174.0 mg). Freeze drying afforded product **24** as a colorless solid (122 mg, 95%).

$[\alpha]_D^{20}$ = +32.6 (c 0.8, $CHCl_3$)

d.r = > 98:2; determined via ^{1}H-NMR and HPLC [HPLC conditions: Chiralcel ® IA column, *n*-hexane/ethanol/DEA = 1:1:0.1, flow rate = 0.7 mL/min, t_R (minor) = 5.1 min and t_R (major) = 7.6 min].

m.p. 87-88 °C

^{1}H NMR (500 MHz, DMSO-d$_6$) δ = 7.23 – 7.13 (m, 5H), 5.75 (s, 1H), 4.98 (d, 1H, *J* = 4 Hz), 2.87 (s, 2H), 2.45 (s, 3H), 2.37 (s, 3H), 2.01 (s, 3H), 1.45 (s, 6H).

^{13}C NMR (126 MHz, DMSO-d$_6$) δ = 171.1, 167.4, 133.6, 129.3, 128.7, 128.0, 57.8, 55.2, 51.8, 29.9, 18.9, 18.2.

MS (APCI) m/z calcd for $C_{21}H_{25}NO_5S$ 404.5 $[M+H]^+$, found 404.3 $[M+H]^+$

2.2.11

(S)-2-(2-chlorophenyl)-2-((2,2,4,6,7-pentamethyl-2,3-dihydrobenzofuran)-5-sulfonamido)acetic acid **25a**

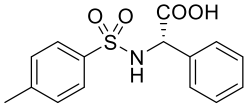

25a

Prepared according to **GP7** from 4-methylbenzenesulfonamide (171,2 mg, 0.50 mmol, 1.0 equiv). Purification by reverse phase column chromatography (H_2O/MeCN + 0.1 vol% TFA = 9:1 → 2:8) and freeze drying afterwards afforded product **25a** as a colorless solid (80 mg, 52%).

$[\alpha]_D^{20}$ = +111.2 (c 0.1, $CHCl_3$)

e.r = 97:3 [HPLC conditions: Chiralcel ® IA column, *n*-hexane/ethanol/TFA = 19:1:0.1, flow rate = 0.7 mL/min, t_R (minor) = 39.4 min and t_R (major) = 41.7 min].

R_f (*n*-hexane/acetone/AcOH = 2:1:0.1) 0.33

m.p. 176-178 °C

^{1}H NMR (400 MHz, DMSO-d_6) δ = 7.23 – 7.13 (m, 5H), 5.75 (s, 1H), 4.98 (d, 1H, *J* = 4 Hz), 2.87 (s, 2H), 2.45 (s, 3H), 2.37 (s, 3H), 2.01 (s, 3H), 1.45 (s, 6H).

^{13}C NMR (101 MHz, DMSO-d_6) δ = 174.92, 159.97, 139.64, 134.93, 134.25, 128.72, 128.64, 127.78, 127.27, 125.16, 118.11, 86.98, 59.13, 43.15, 28.66, 28.64, 19.39, 17.77, 12.50.

MS (APCI) m/z calcd for $C_{15}H_{15}NO_4S$ 306.1 $[M+H]^+$, found 306.4 $[M+H]^+$

HRMS (TOF MS EI+) m/z calcd for $C_{15}H_{15}NO_4S$ 305.0722 $[M^+]$, found 305.0713 $[M^+]$

IR (v in cm^{-1}): 3585, 3528, 3319, 3292, 2970, 1725, 1691, 1598, 1459, 1344, 1325, 1164, 1088, 921, 896, 812, 686, 529.

2.2.12

(S)-2-phenyl-2-((2,4,6-triisopropylphenyl)sulfonamido)acetic acid **27a**

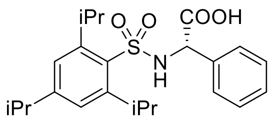

27a

Prepared according to **GP7** from 2,4,6-triisopropylphenylsulfonamide (141.7 mg, 0.50 mmol, 1.0 equiv). Purification by reverse phase column chromatography (H_2O/MeCN + 0.1 vol% TFA = 9:1 → 2:8) and freeze drying afterwards afforded product **27a** as a colorless solid (153 mg, 73%).

$[\alpha]_D^{20}$ = 0.0 (c 0.1, $CHCl_3$)

R_f (*n*-hexane/acetone/AcOH = 1:1:0.1) 0.73

m.p. 117-119 °C

^{1}H NMR (400 MHz, DMSO-d_6) δ = 8.57 (d, *J* = 8 Hz, 1H), 7.29 – 7.25 (m, 5H), 7.16 (s, 2H), 4.80 (d, *J* = 8 Hz, 1H), 4.11 (quin, *J* = 4 Hz, 2H), 2.88 (quin, *J* = 8 Hz, 1H), 1.18 (t, *J* = 8 Hz, 12H), 1.11 (s, 6H).

MS (APCI) m/z calcd for $C_{23}H_{31}NO_4S$ 416.19 $[M-H]^-$, found 416.10 $[M-H]^-$

IR (v in cm^{-1}): 3182, 2959, 1702, 1456, 1257, 1164, 1070, 881, 697, 662, 561.

3 HPLC Data

3.1 (S)-2-((2,2,4,6,7-pentamethyl-2,3-dihydrobenzofuran)-5-sulfonamido)-2-phenylacetic acid **13a**

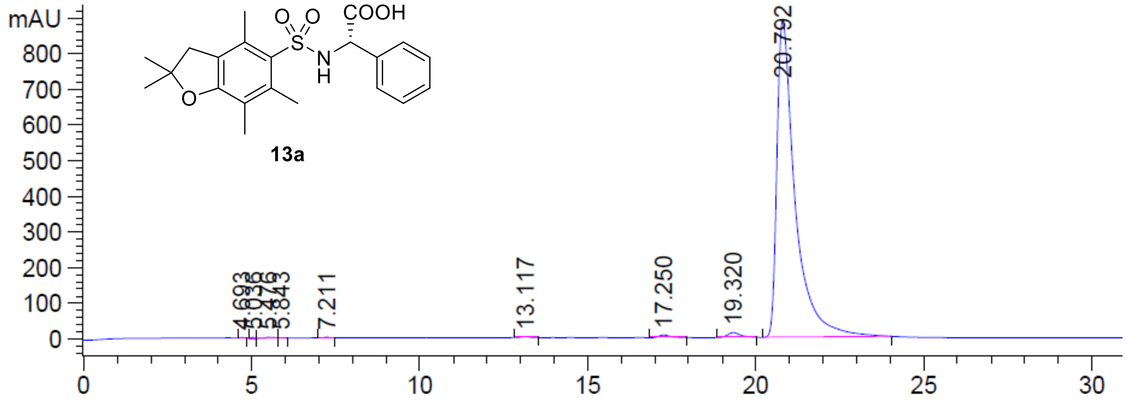

Signal 2: DAD1 B, Sig=254,16 Ref=380,100

Peak #	RetTime [min]	Type	Width [min]	Area [mAU*s]	Height [mAU]	Area %
1	4.693	BB	0.1476	22.24450	2.03384	0.0647
2	5.036	BV	0.0941	27.72850	4.36556	0.0807
3	5.476	VV	0.2800	89.24469	4.00485	0.2596
4	5.843	VB	0.1483	17.29930	1.57274	0.0503
5	7.211	BB	0.1524	22.21033	2.05078	0.0646
6	13.117	BB	0.2592	51.31150	2.97458	0.1493
7	17.250	BB	0.3492	176.58760	7.53997	0.5137
8	19.320	BB	0.4111	377.58954	13.80683	1.0985
9	20.792	BB	0.5520	3.35900e4	886.52686	97.7186

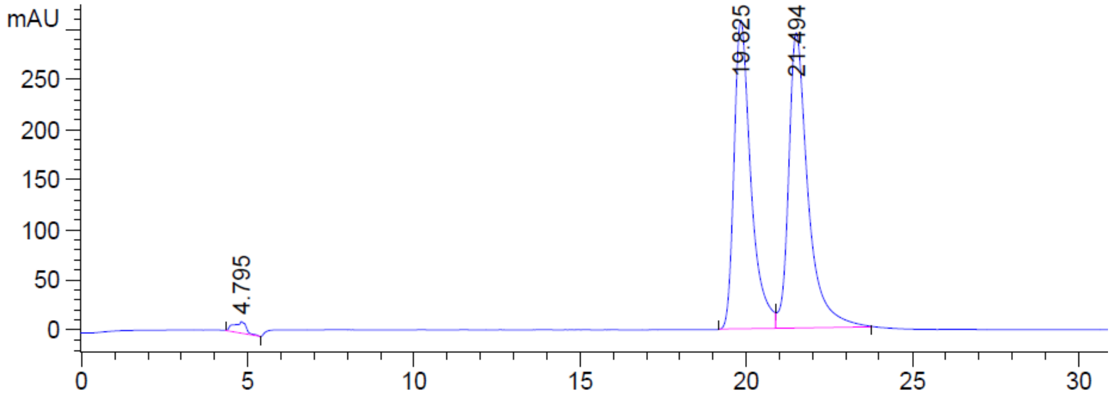

Peak #	RetTime [min]	Type	Width [min]	Area [mAU*s]	Height [mAU]	Area %
1	4.795	BV	0.3316	307.34262	11.65567	1.3259
2	19.825	BV	0.5289	1.09077e4	308.19196	47.0582
3	21.494	VB	0.6017	1.19642e4	294.68765	51.6158

3.2 (S)-2-((2,2,4,6,7-pentamethyl-2,3-dihydrobenzofuran)-5-sulfonamido)-2-(p-tolyl)acetic acid **14a**

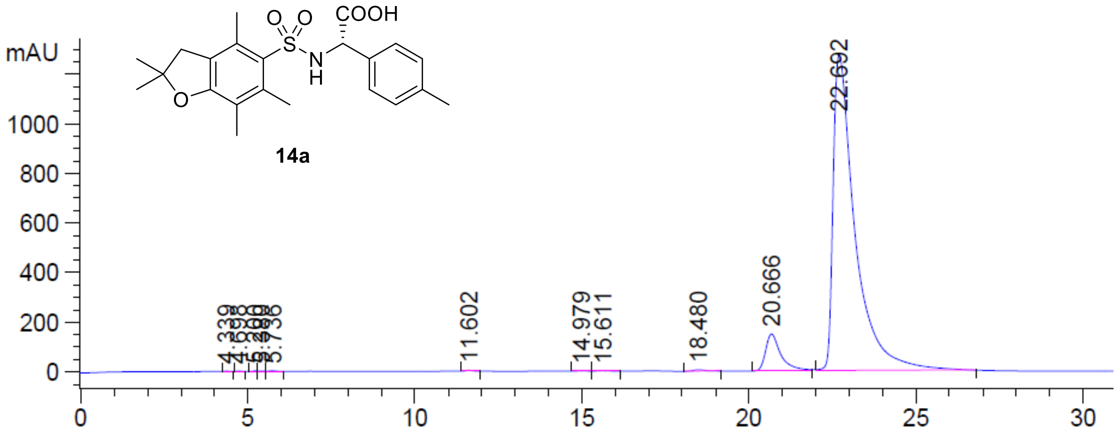

Peak #	RetTime [min]	Type	Width [min]	Area [mAU*s]	Height [mAU]	Area %
1	4.339	BB	0.1652	18.08113	1.51578	0.0280
2	4.698	BB	0.1311	20.25197	2.12594	0.0313
3	5.200	BV	0.0903	19.42182	3.13390	0.0301
4	5.380	VV	0.1801	18.58668	1.64187	0.0288
5	5.736	VB	0.1736	40.73038	3.17617	0.0630
6	11.602	BB	0.2215	52.06084	3.46156	0.0806
7	14.979	BV	0.2611	33.93132	1.80674	0.0525
8	15.611	VB	0.3131	46.09687	1.98392	0.0713
9	18.480	BB	0.3866	157.09587	5.82499	0.2431
10	20.666	BB	0.4691	4806.25488	149.48354	7.4382
11	22.692	BB	0.6609	5.84696e4	1275.24658	90.4880
12	35.806	BB	0.5325	933.75098	24.28960	1.4451

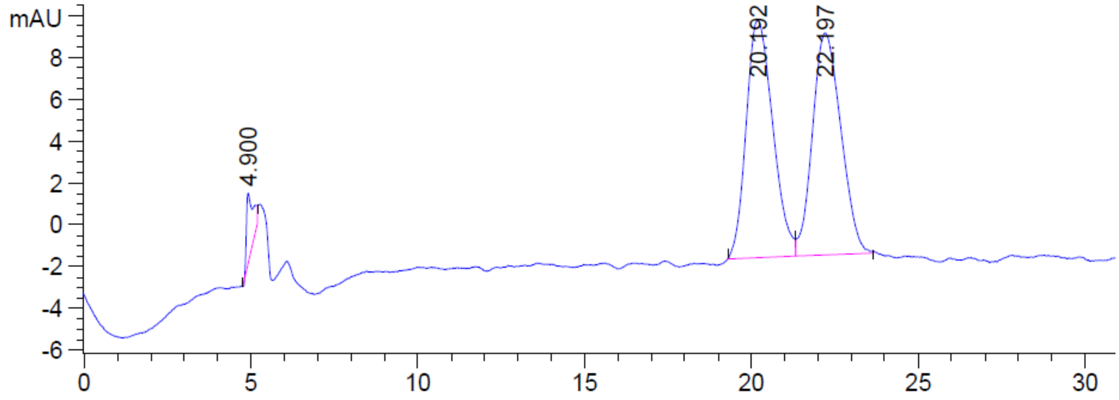

Signal 2: DAD1 B, Sig=254,16 Ref=380,100

Peak #	RetTime [min]	Type	Width [min]	Area [mAU*s]	Height [mAU]	Area %
1	4.900	BB	0.1789	44.67887	3.40936	3.4088
2	20.192	BV	0.6723	633.50110	11.36460	48.3327
3	22.197	VB	0.7197	632.53033	10.63719	48.2586

3.3 (S)-2-(4-fluorophenyl)-2-((2,2,4,6,7-pentamethyl-2,3-dihydrobenzofuran)-5-sulfonamido)acetic acid **15a**

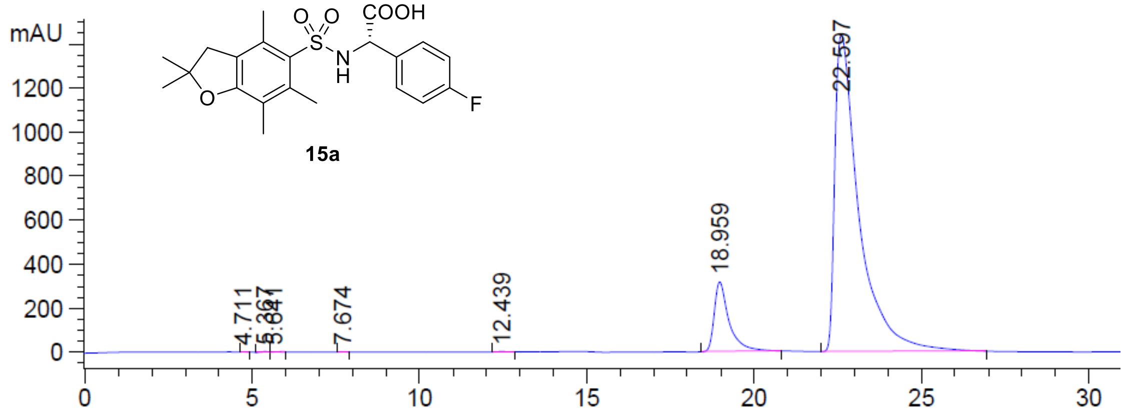

Signal 2: DAD1 B, Sig=254,16 Ref=380,100

Peak #	RetTime [min]	Type	Width [min]	Area [mAU*s]	Height [mAU]	Area %
1	4.711	BB	0.0935	9.36246	1.48555	0.0113
2	5.367	BV	0.2016	69.08786	4.59024	0.0837
3	5.641	VB	0.2017	44.87453	2.88103	0.0544
4	7.674	BB	0.1471	9.87983	1.00185	0.0120
5	12.439	BB	0.2354	31.18032	1.92092	0.0378
6	18.959	BB	0.4547	9845.96387	316.70044	11.9325
7	22.597	BB	0.7366	7.25036e4	1437.13098	87.8683

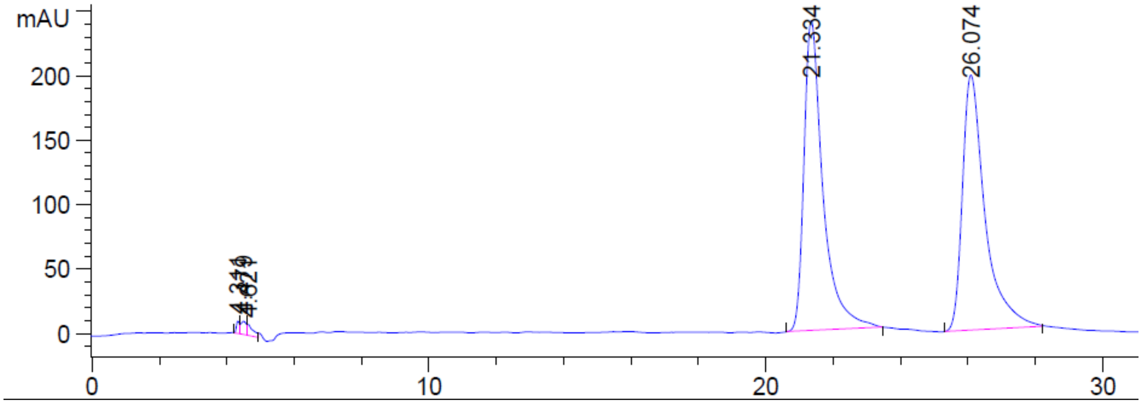

Signal 2: DAD1 B, Sig=254,16 Ref=380,100

Peak #	RetTime [min]	Type	Width [min]	Area [mAU*s]	Height [mAU]	Area %
1	4.311	BV	0.1077	69.33904	9.89981	0.3646
2	4.479	VV	0.1678	119.82873	10.60920	0.6301
3	4.621	VB	0.1634	107.53255	8.74918	0.5654
4	21.334	BB	0.5805	9498.18066	240.64174	49.9418
5	26.074	BB	0.6964	9223.63574	197.66888	48.4982

3.4 (S)-2-(4-chlorophenyl)-2-((2,2,4,6,7-pentamethyl-2,3-dihydrobenzofuran)-5-sulfonamido)acetic acid **16a**

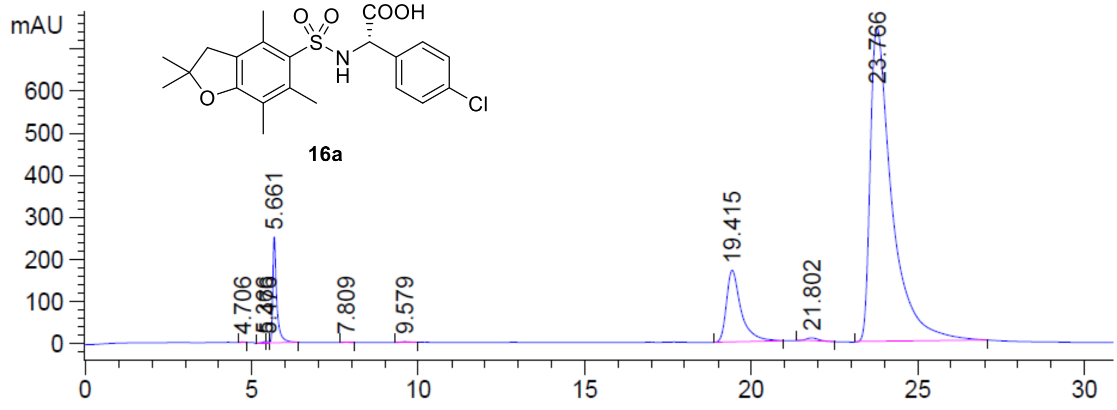

Signal 2: DAD1 B, Sig=254,16 Ref=380,100

Peak #	RetTime [min]	Type	Width [min]	Area [mAU*s]	Height [mAU]	Area %
1	4.706	BB	0.0934	9.04155	1.47813	0.0217
2	5.366	BV	0.1389	40.88613	4.01086	0.0982
3	5.476	VV	0.0852	23.45727	3.95210	0.0563
4	5.661	VB	0.1186	2094.95142	253.00662	5.0299
5	7.809	BB	0.1500	10.77649	1.06554	0.0259
6	9.579	BB	0.2678	30.13076	1.65984	0.0723
7	19.415	BB	0.4496	5189.18408	170.23573	12.4590
8	21.802	BB	0.4055	206.49976	7.63524	0.4958
9	23.766	BB	0.6715	3.40452e4	747.26428	81.7409

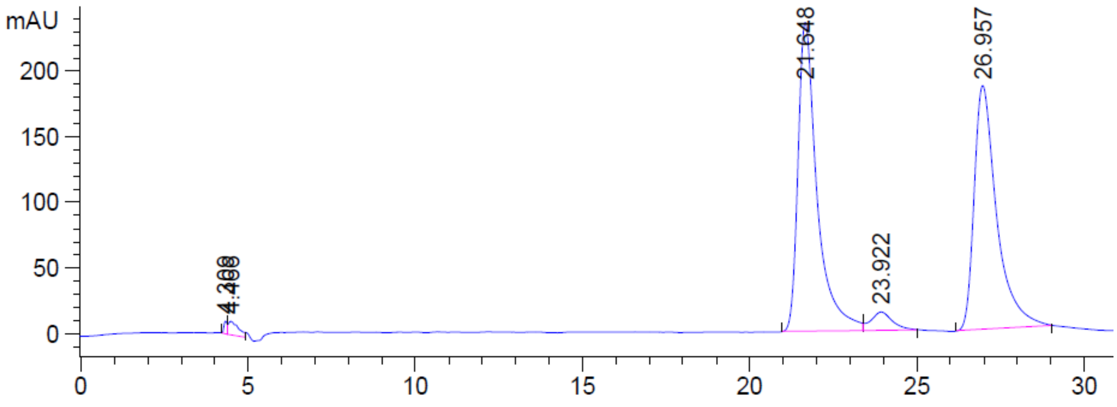

Signal 2: DAD1 B, Sig=254,16 Ref=380,100

Peak #	RetTime [min]	Type	Width [min]	Area [mAU*s]	Height [mAU]	Area %
1	4.309	BV	0.1083	68.42589	9.70096	0.3593
2	4.466	VB	0.2637	215.05797	10.29743	1.1294
3	21.648	BB	0.5898	9361.87988	235.47879	49.1641
4	23.922	BB	0.6231	633.47345	14.07428	3.3267
5	26.957	BB	0.7057	8763.26855	186.00394	46.0205

3.5 (S)-2-(4-methoxyphenyl)-2-((2,2,4,6,7-pentamethyl-2,3-dihydrobenzofuran)-5-sulfonamido)acetic acid **17a**

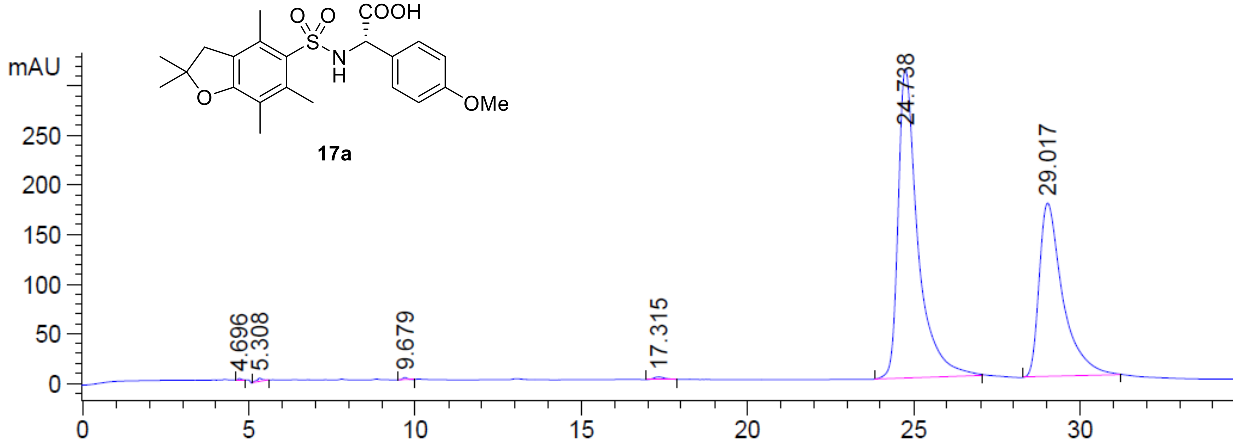

Signal 2: DAD1 B, Sig=254,16 Ref=380,100

Peak #	RetTime [min]	Type	Width [min]	Area [mAU*s]	Height [mAU]	Area %
1	4.696	BB	0.0935	10.55730	1.63154	0.0486
2	5.308	BB	0.1923	54.25526	3.80694	0.2497
3	9.679	BB	0.1673	26.48218	2.35345	0.1219
4	17.315	BB	0.3237	60.08017	2.65793	0.2765
5	24.738	BB	0.6062	1.29057e4	312.22900	59.3872
6	29.017	BB	0.7333	8674.36230	175.30928	39.9163

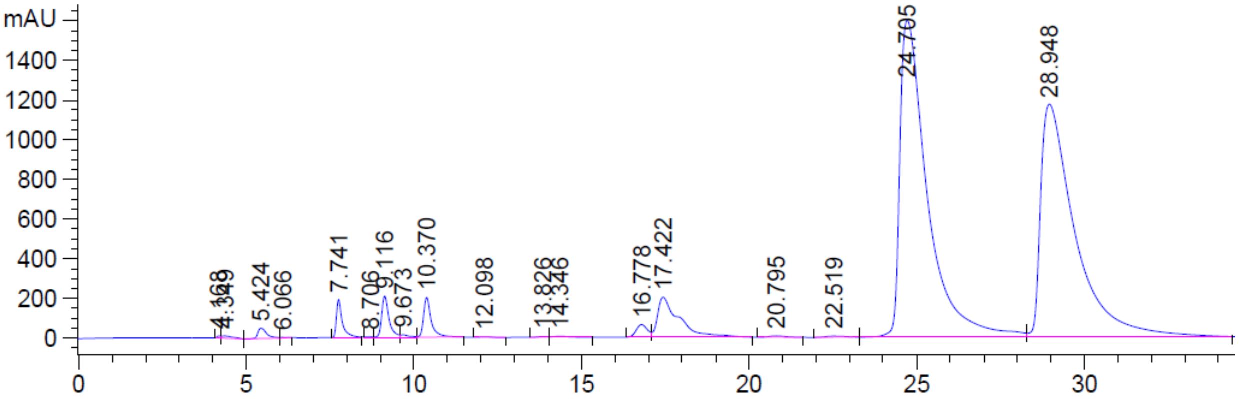

Signal 2: DAD1 B, Sig=254,16 Ref=380,100

Peak #	RetTime [min]	Type	Width [min]	Area [mAU*s]	Height [mAU]	Area %
1	4.168	BV	0.1132	82.02338	11.23207	0.0415
2	4.349	VV	0.3077	305.31033	12.92062	0.1547
3	5.424	VB	0.2885	992.92761	52.53289	0.5030
4	6.066	BB	0.1972	55.68750	3.75167	0.0282
5	7.741	BB	0.1814	2395.23901	192.18964	1.2133
6	8.706	BV	0.2108	58.75045	3.92189	0.0298
7	9.116	VV	0.2266	3200.89844	209.14458	1.6214
8	9.673	VV	0.2479	232.84340	13.06846	0.1179
9	10.370	VB	0.2382	3289.87109	201.78976	1.6665
10	12.098	BB	0.2589	46.99249	2.60005	0.0238
11	13.826	BV	0.3355	92.35009	3.80041	0.0468
12	14.346	VB	0.4744	196.36867	5.75023	0.0995
13	16.778	BV	0.3580	1479.89856	63.95156	0.7497
14	17.422	VB	0.6206	9127.63574	202.22566	4.6237
15	20.795	BB	0.5354	270.13916	7.47817	0.1368
16	22.519	BV	0.5264	277.62607	6.32148	0.1406
17	24.705	VV	0.8434	9.15133e4	1599.83997	46.3570
18	28.948	VB	1.0315	8.37920e4	1175.74207	42.4457

3.6 (S)-2-(3-chlorophenyl)-2-((2,2,4,6,7-pentamethyl-2,3-dihydrobenzofuran)-5-sulfonamido)acetic acid **18a**

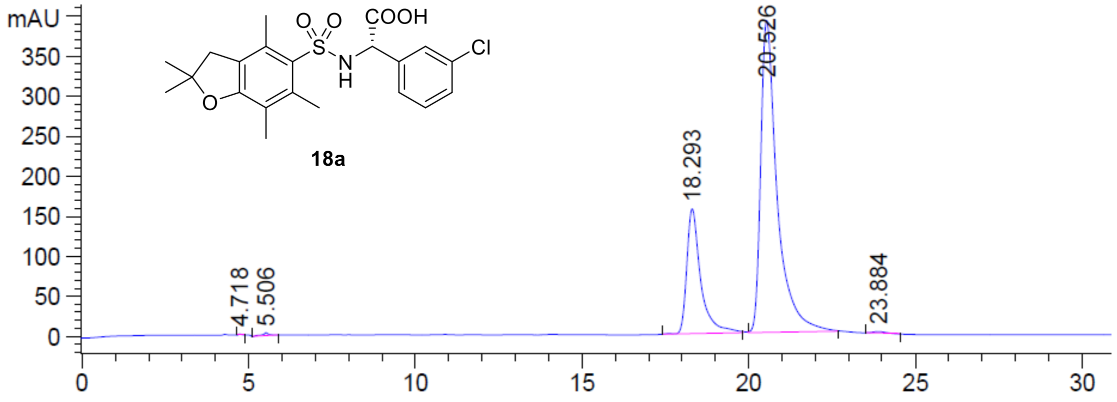

Signal 2: DAD1 B, Sig=254,16 Ref=380,100

Peak #	RetTime [min]	Type	Width [min]	Area [mAU*s]	Height [mAU]	Area %
1	4.718	BB	0.0927	9.25484	1.48470	0.0511
2	5.506	BB	0.2204	69.81905	4.10569	0.3853
3	18.293	BB	0.4310	4589.45166	156.08591	25.3302
4	20.526	BB	0.5071	1.33879e4	389.38815	73.8906
5	23.884	BB	0.3618	62.11916	2.07170	0.3428

3.7 ((S)-2-((2,2,4,6,7-pentamethyl-2,3-dihydrobenzofuran)-5-sulfonamido)-2-(o-tolyl)acetic acid **19a**

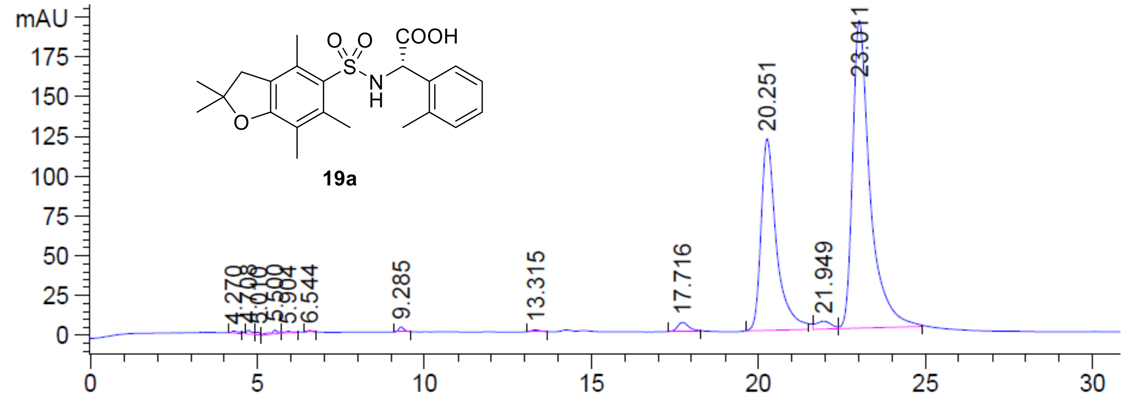

Signal 2: DAD1 B, Sig=254,16 Ref=380,100

Peak #	RetTime [min]	Type	Width [min]	Area [mAU*s]	Height [mAU]	Area %
1	4.270	BB	0.2064	20.98210	1.38756	0.1824
2	4.708	BB	0.1700	34.09470	2.68765	0.2963
3	5.010	BV	0.1017	13.36413	1.95661	0.1162
4	5.500	VV	0.2362	50.51608	2.75144	0.4391
5	5.904	VB	0.2294	23.05769	1.30968	0.2004
6	6.544	BB	0.1183	8.74047	1.10429	0.0760
7	9.285	BB	0.1807	37.61091	3.16251	0.3269
8	13.315	BB	0.2430	21.85636	1.25619	0.1900
9	17.716	BB	0.3618	137.96373	5.71077	1.1991
10	20.251	BB	0.4766	3916.44604	120.69061	34.0402
11	21.949	BV	0.4234	161.38660	5.04302	1.4027
12	23.011	VB	0.5387	7079.34717	193.54718	61.5308

3.8 (S)-2-(2-chlorophenyl)-2-((2,2,4,6,7-pentamethyl-2,3-dihydrobenzofuran)-5-sulfonamido)acetic acid **20a**

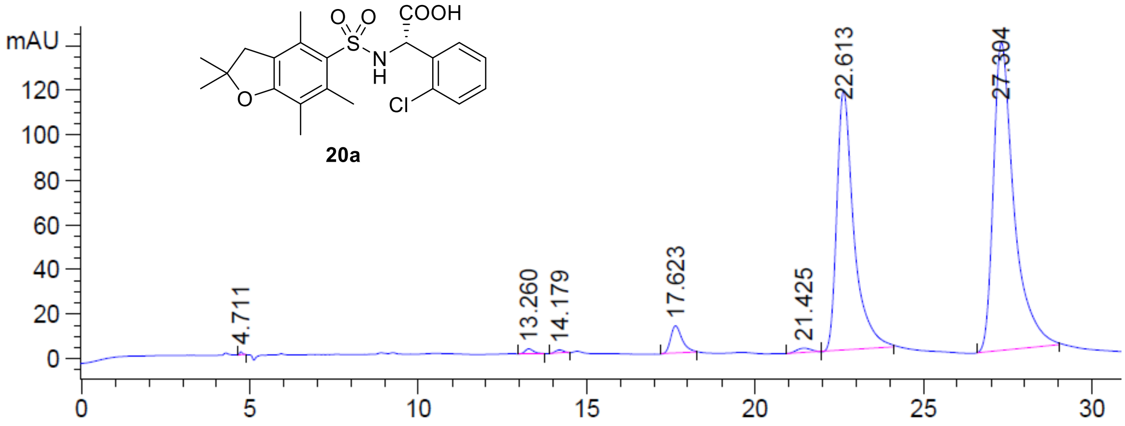

Signal 2: DAD1 B, Sig=254,16 Ref=380,100

Peak #	RetTime [min]	Type	Width [min]	Area [mAU*s]	Height [mAU]	Area %
1	4.711	BB	0.0916	8.90109	1.45072	0.0854
2	13.260	BB	0.2575	39.63025	2.25028	0.3802
3	14.179	BV	0.2386	25.51202	1.49885	0.2447
4	17.623	BB	0.3661	298.35864	12.33883	2.8622
5	21.425	BV	0.3790	64.51175	2.07320	0.6189
6	22.613	VB	0.5212	4093.14819	116.12399	39.2657
7	27.304	BB	0.6334	5894.18213	137.68275	56.5430

3.9 (S)-2-(((S)-1-methoxy-3-methyl-1-oxobutan-2-yl)amino)-2-oxo-1-phenylethan-1-aminium trifluoroacetate **23**

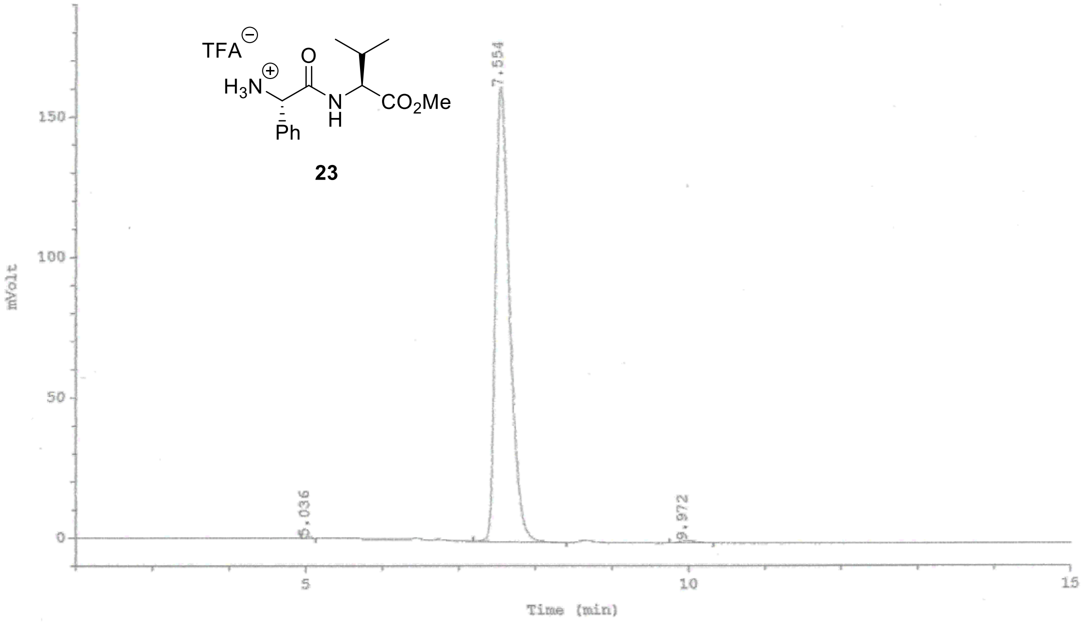

Sample **24** after incubation with base at 40 °C for 12 h.

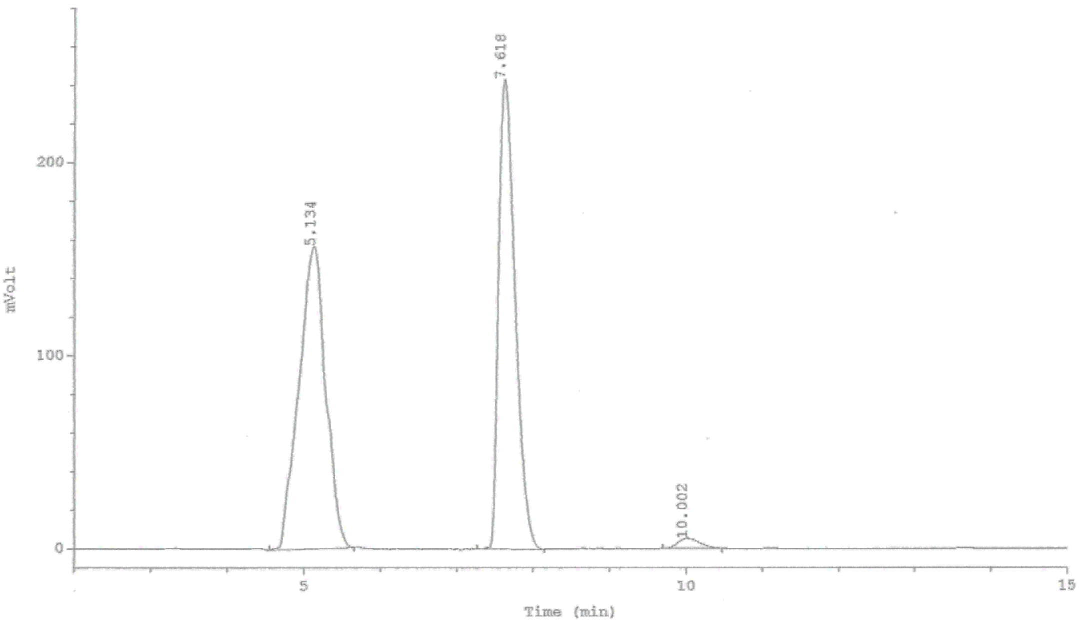

3.10 (S)-2-((4-methylphenyl)sulfonamido)-2-phenylacetic acid **25a**

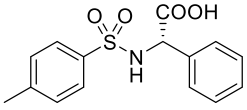

25a

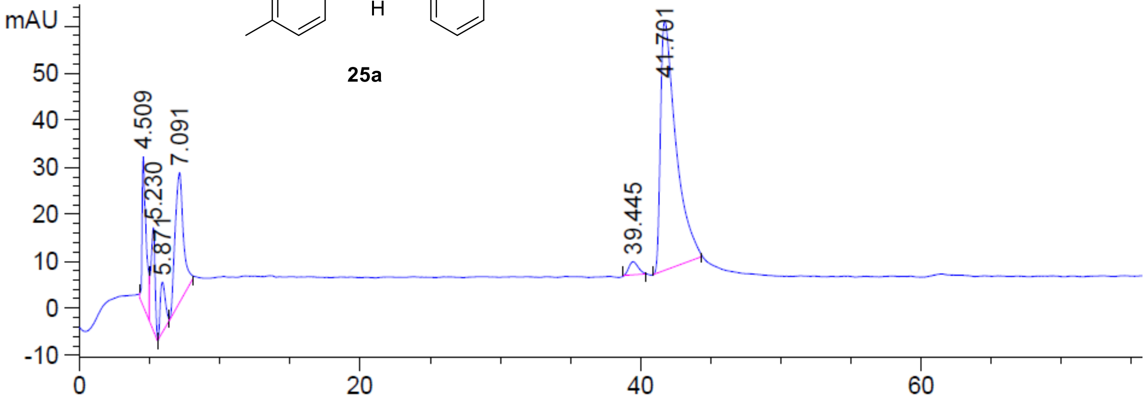

Peak #	RetTime [min]	Type	Width [min]	Area [mAU*s]	Height [mAU]	Area %
1	4.509	BV	0.2864	644.39343	31.80770	9.5639
2	5.230	VV	0.2982	426.10321	21.78304	6.3241
3	5.871	VV	0.3936	275.98572	10.89264	4.0961
4	7.091	VB	0.5993	1165.17419	27.89482	17.2931
5	39.445	BB	0.5606	137.07889	2.91539	2.0345
6	41.701	BB	1.0575	4089.05273	53.15480	60.6884

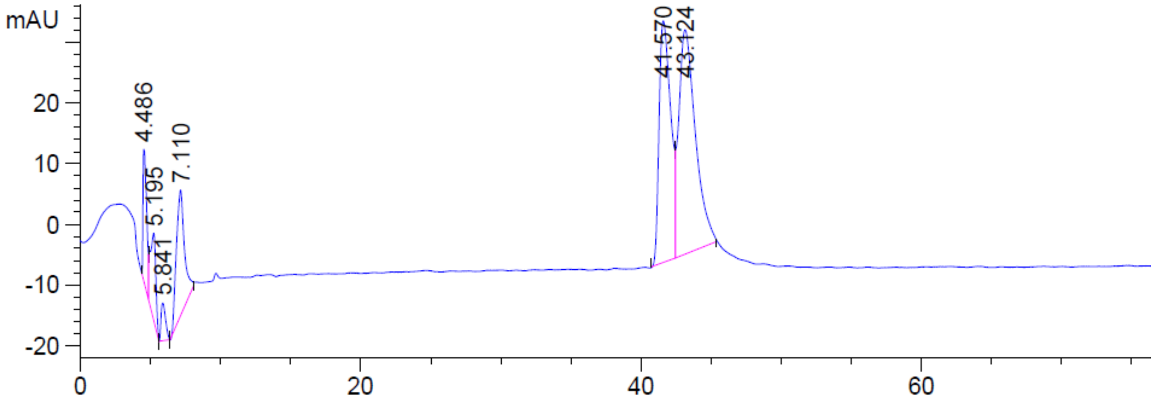

Signal 2: DAD1 B, Sig=254,16 Ref=380,100

Peak #	RetTime [min]	Type	Width [min]	Area [mAU*s]	Height [mAU]	Area %
1	4.486	BV	0.2426	402.74329	21.69717	5.6407
2	5.195	VV	0.3660	379.99963	14.30268	5.3222
3	5.841	VV	0.3353	141.89893	6.24093	1.9874
4	7.110	VB	0.6000	846.10620	20.82493	11.8504
5	41.570	BV	0.8156	2287.25317	39.85336	32.0348
6	43.124	VB	1.1196	3081.89429	37.00745	43.1644

4 NMR Data

4.1 ^{1}H-NMR 13a

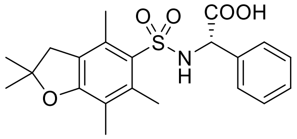

13a

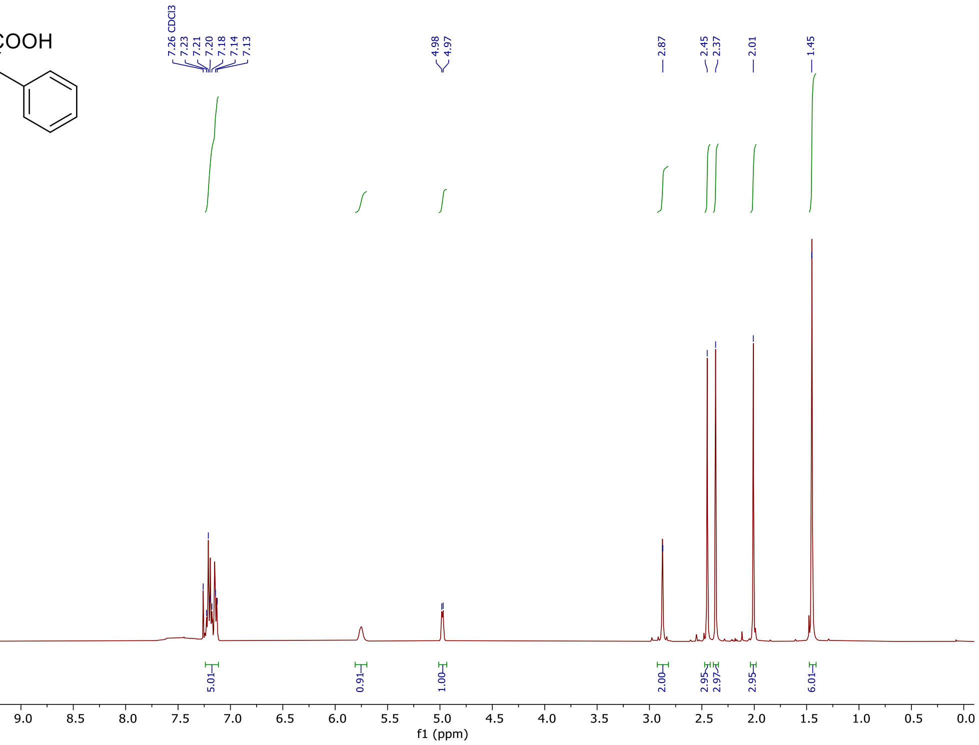

^{13}C-NMR 13a

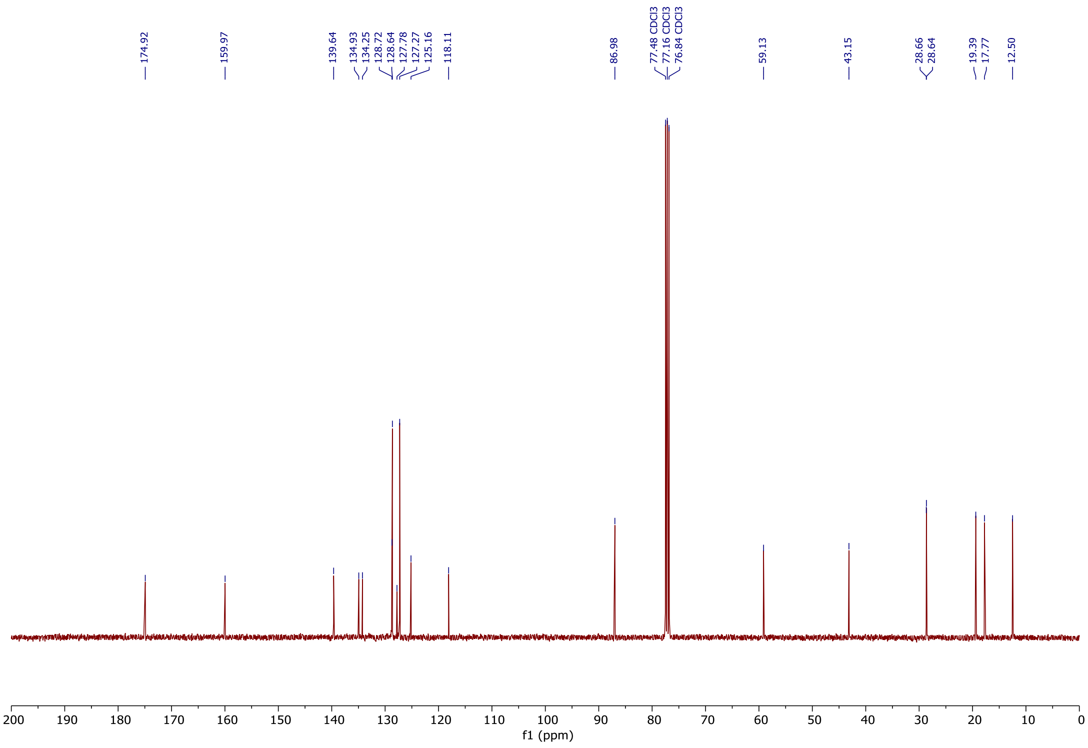

4.2 ^{1}H-NMR 14a

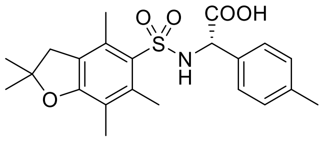

14a

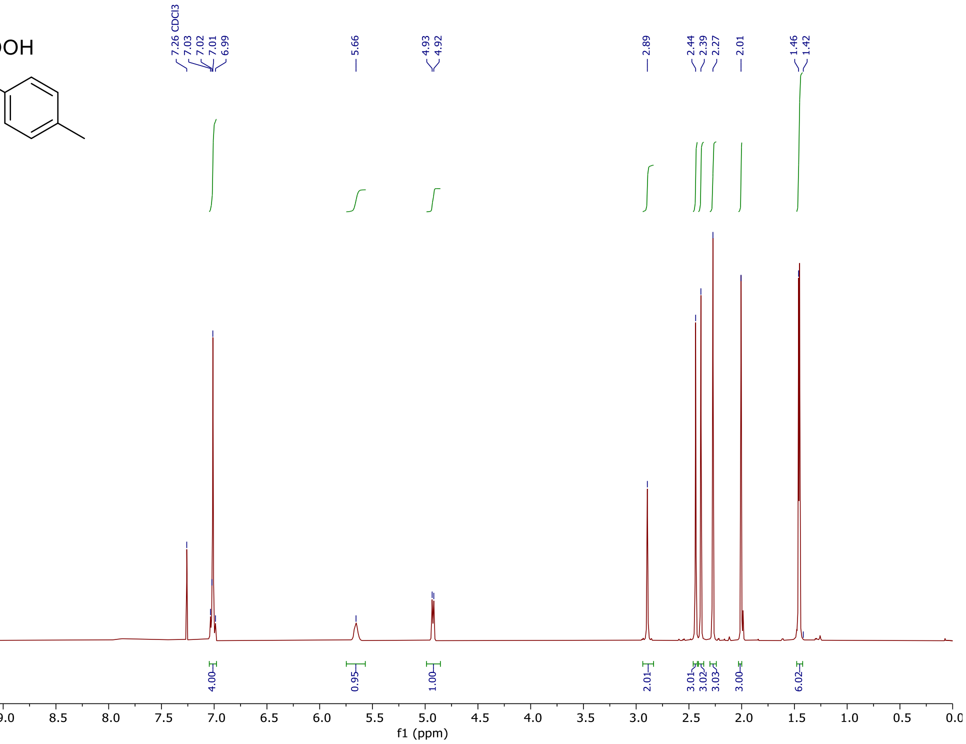

^{13}C-NMR 14a

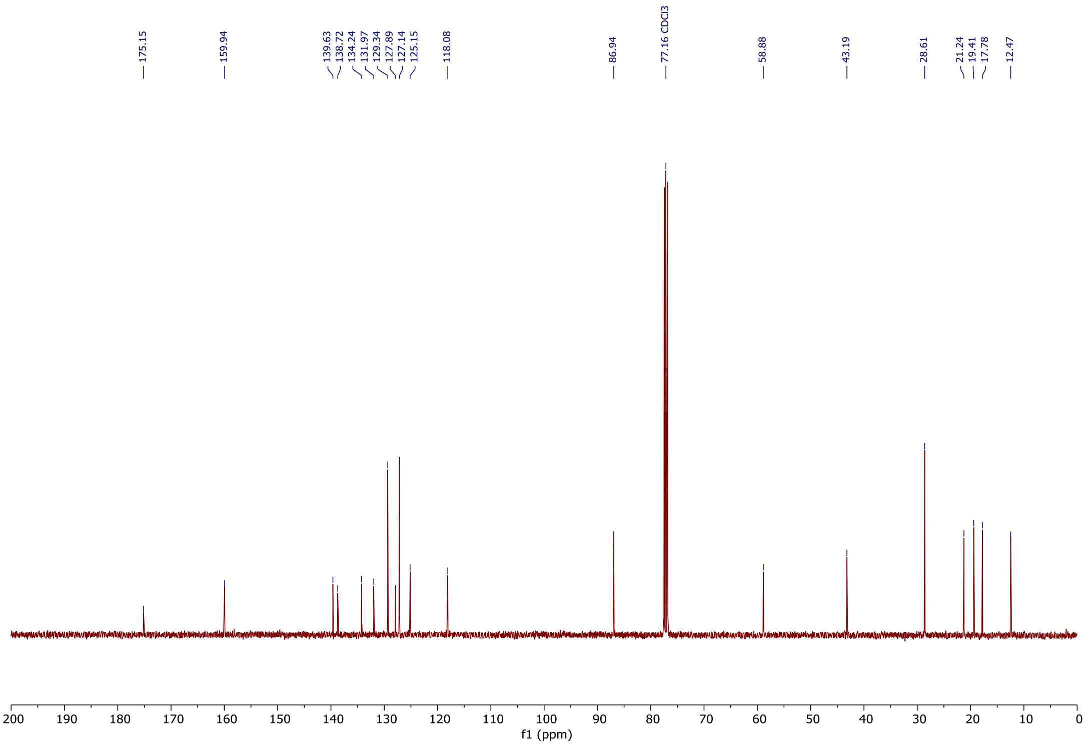

4.3 ^{1}H-NMR 15a

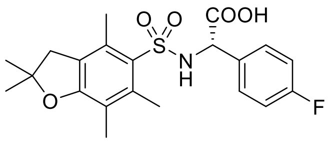

15a

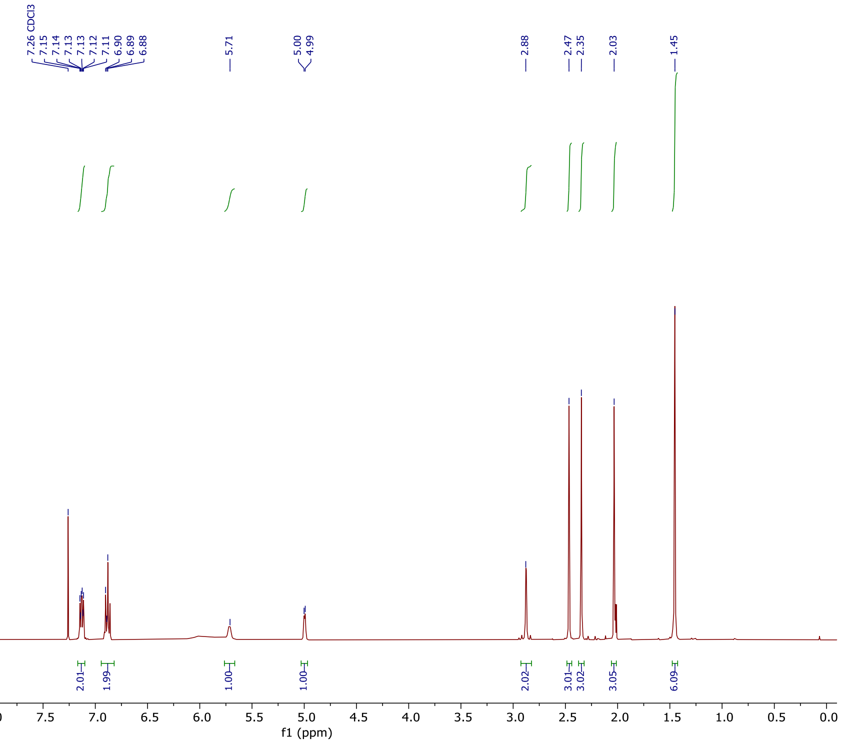

^{13}C-NMR 15a

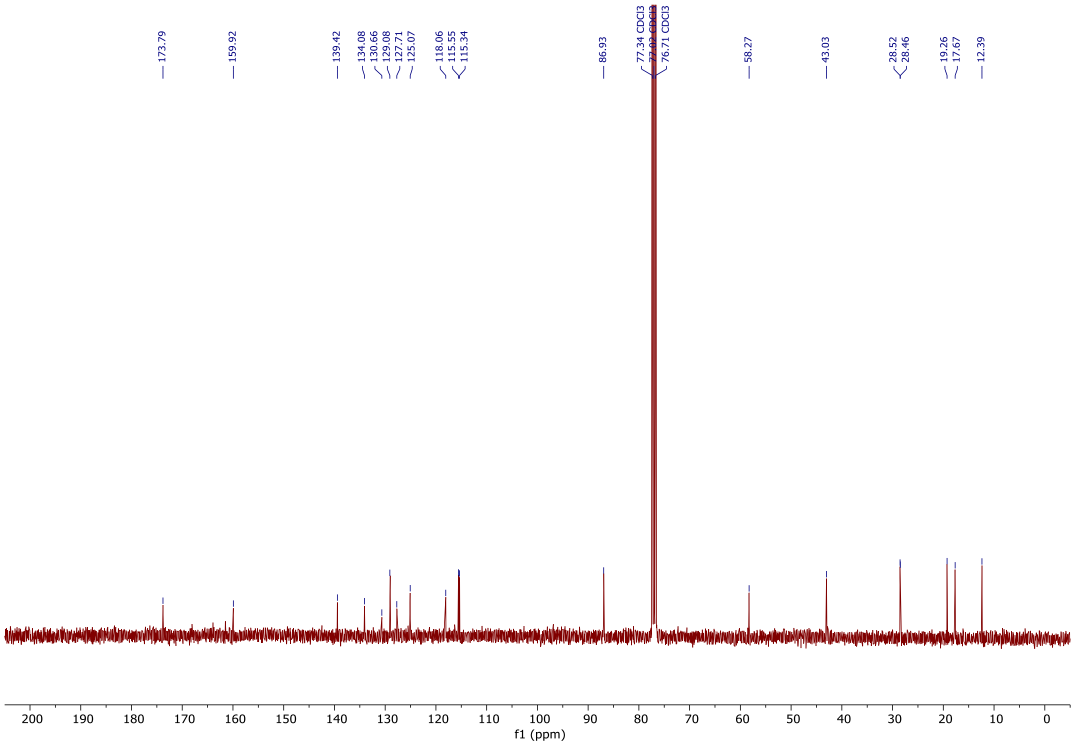

^{19}F-NMR 15a

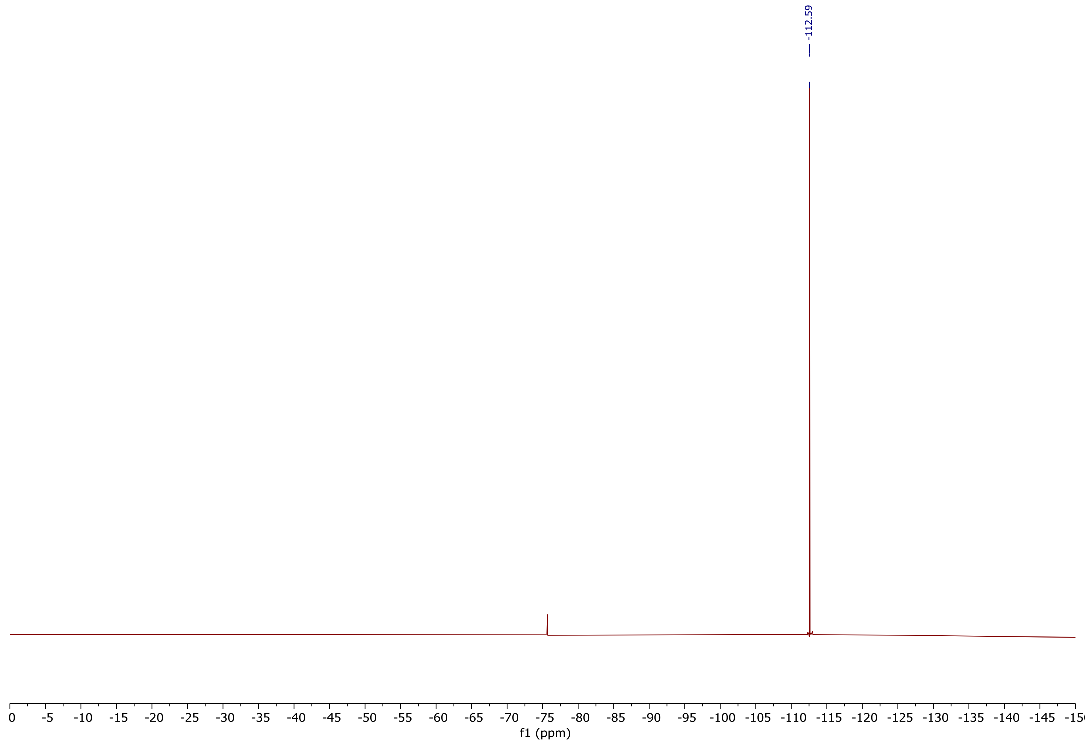

-112.59
0 -5 -10 -15 -20 -25 -30 -35 -40 -45 -50 -55 -60 -65 -70 -75 -80 -85 -90 -95 -100 -105 -110 -115 -120 -125 -130 -135 -140 -145 -150
f1 (ppm)

4.4 ^{1}H-NMR 16a

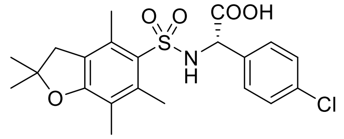

16a

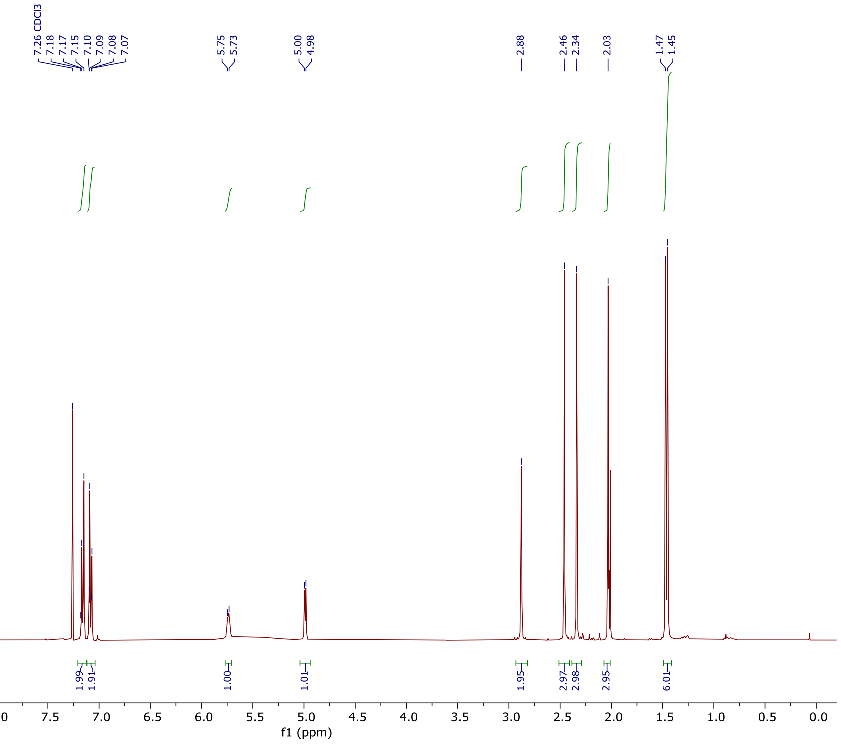

^{13}C-NMR 16a

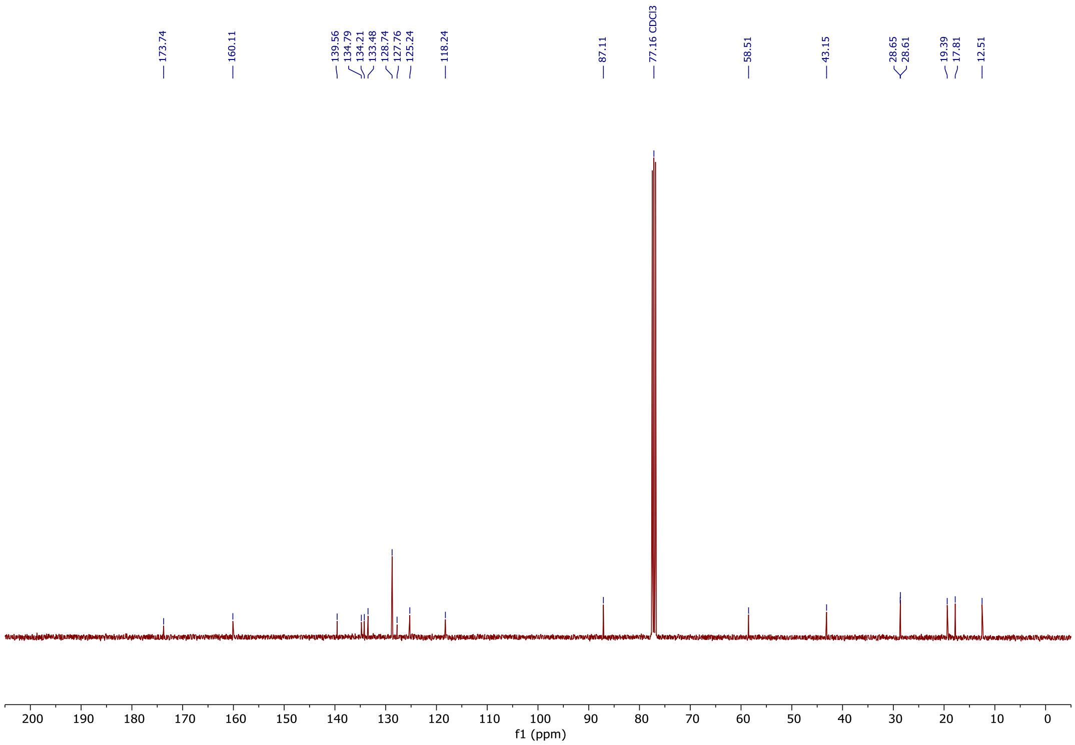

4.5 ^{1}H-NMR 17a

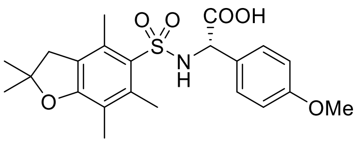

17a

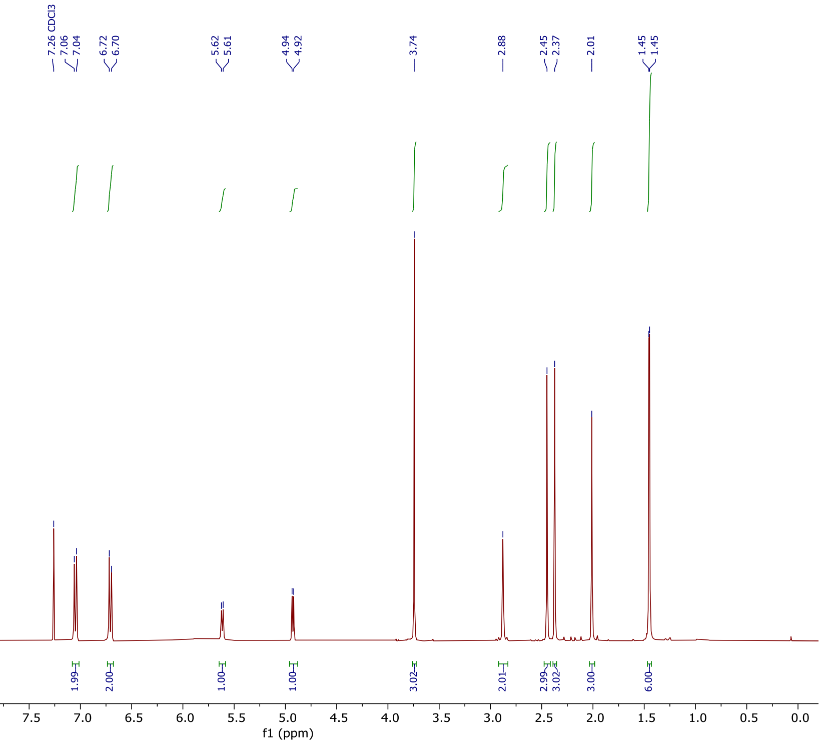

^{13}C-NMR 17a

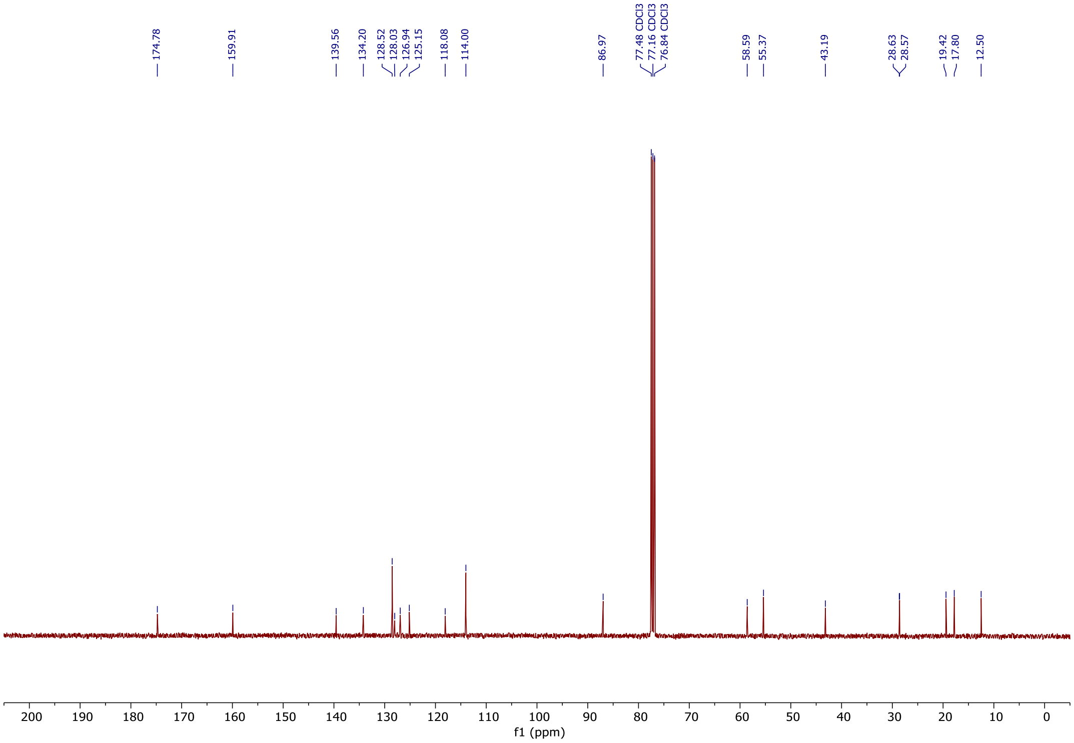

4.6 ^{1}H-NMR 18a

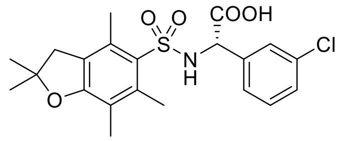

18a

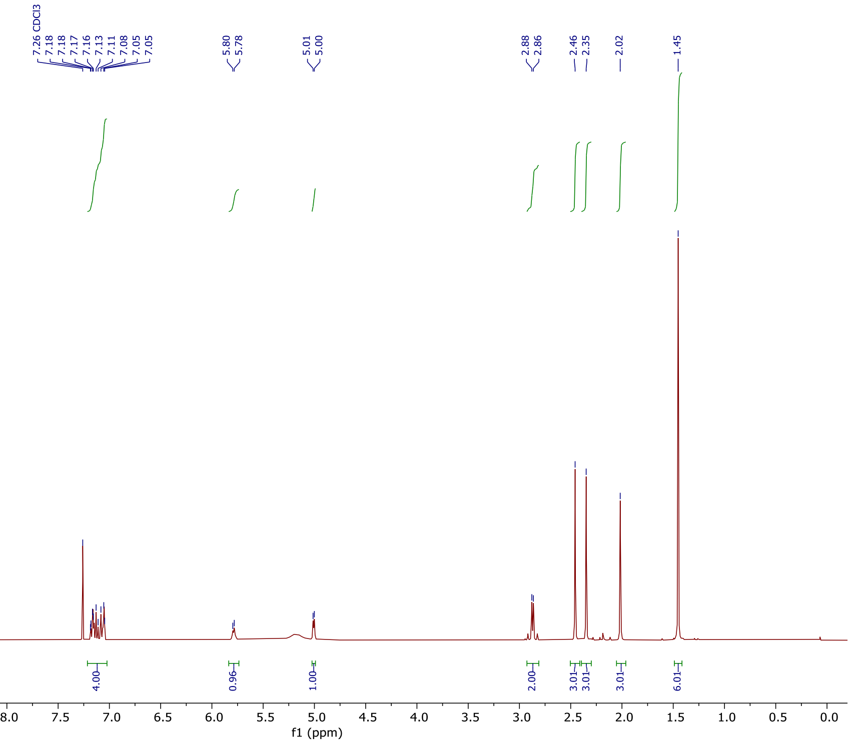

^{13}C-NMR 18a

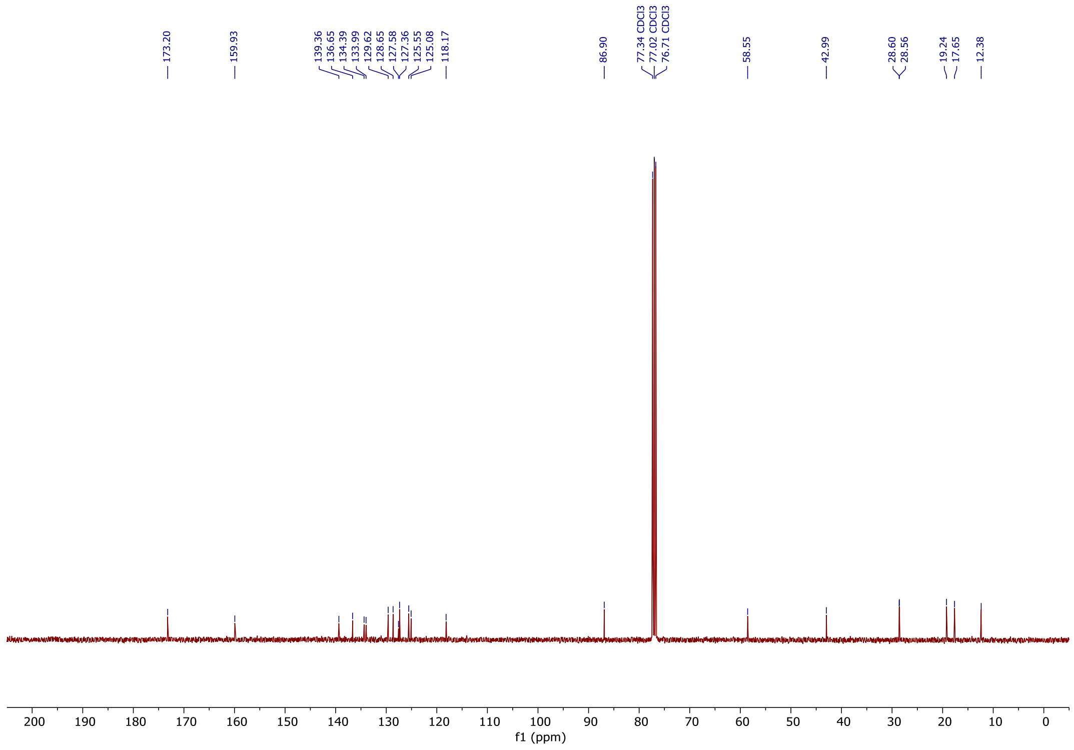

4.7 ^{1}H-NMR 19a

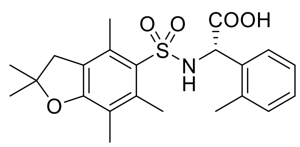

19a

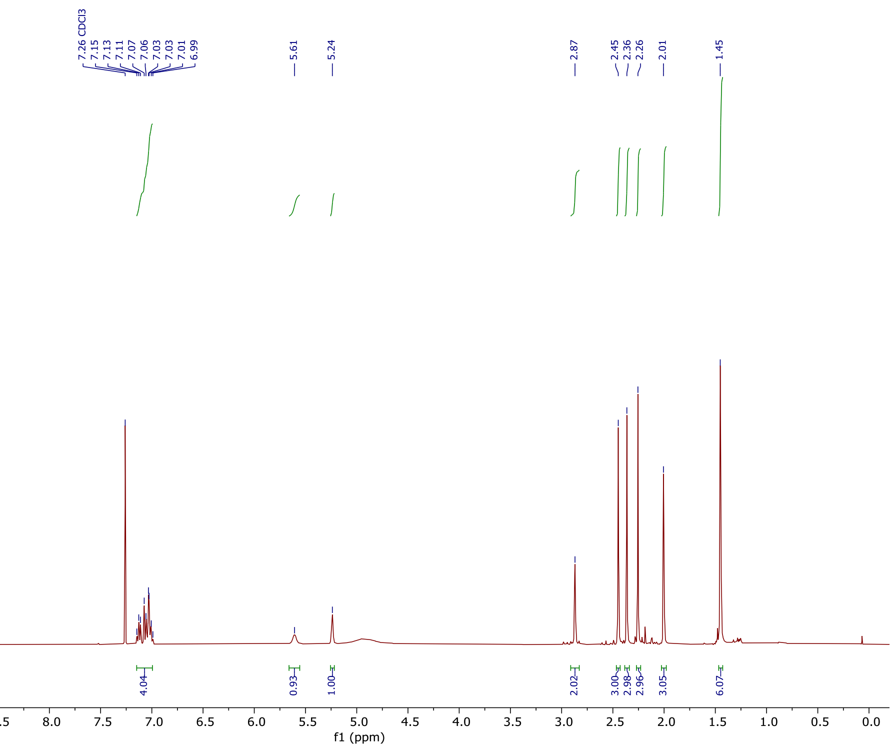

^{13}C-NMR 19a

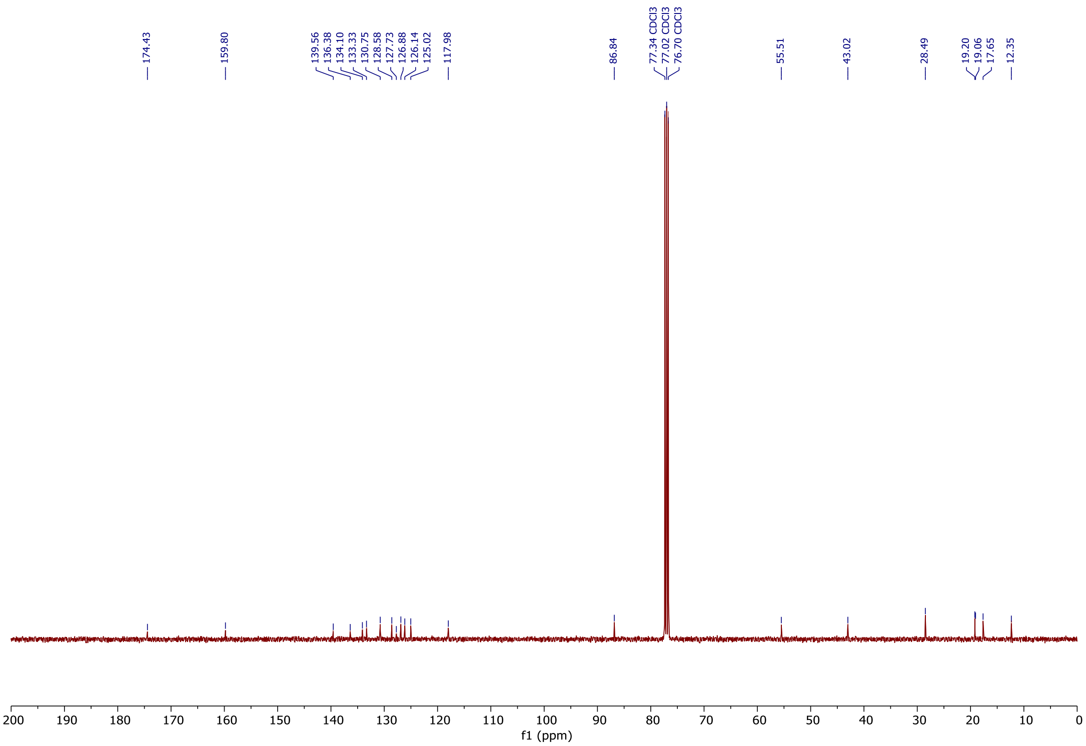

4.8 ^{1}H-NMR 20a

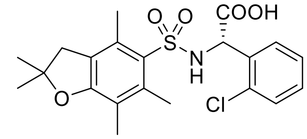

20a

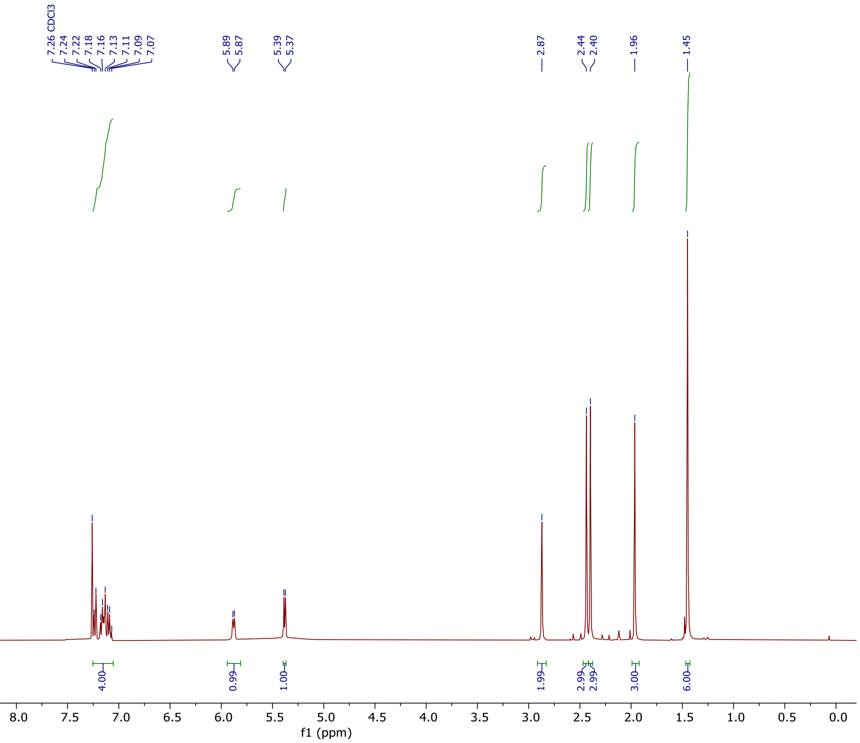

^{13}C-NMR 20a

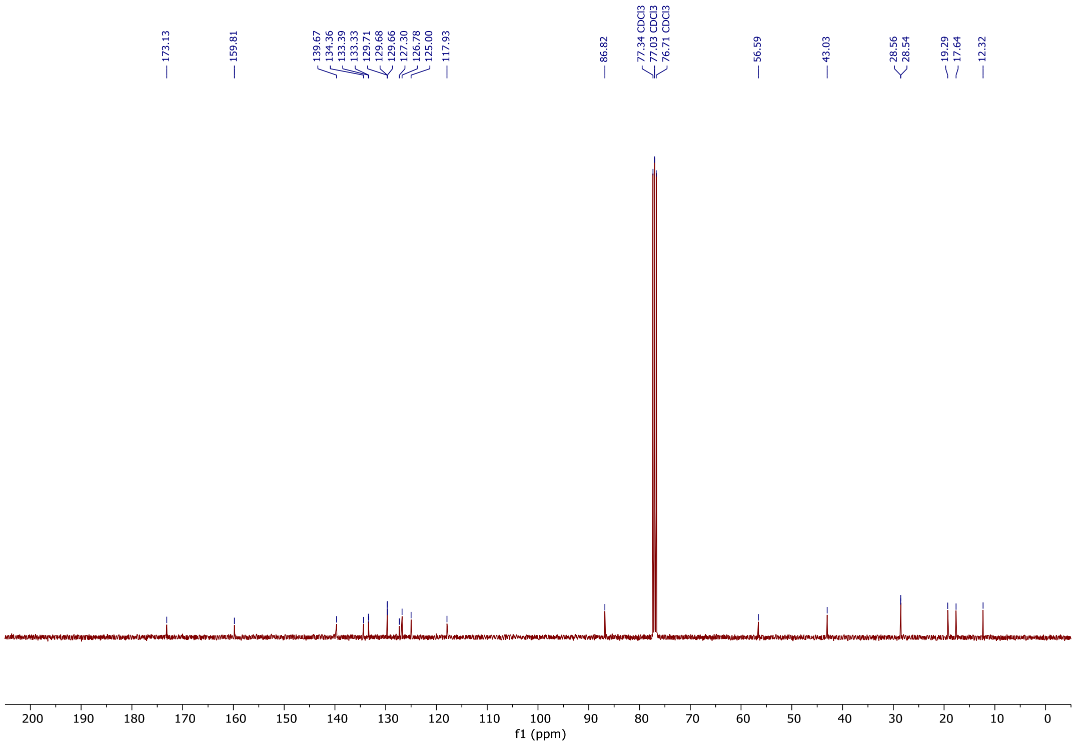

4.9 ^{1}H-NMR 22

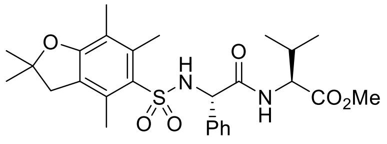

22

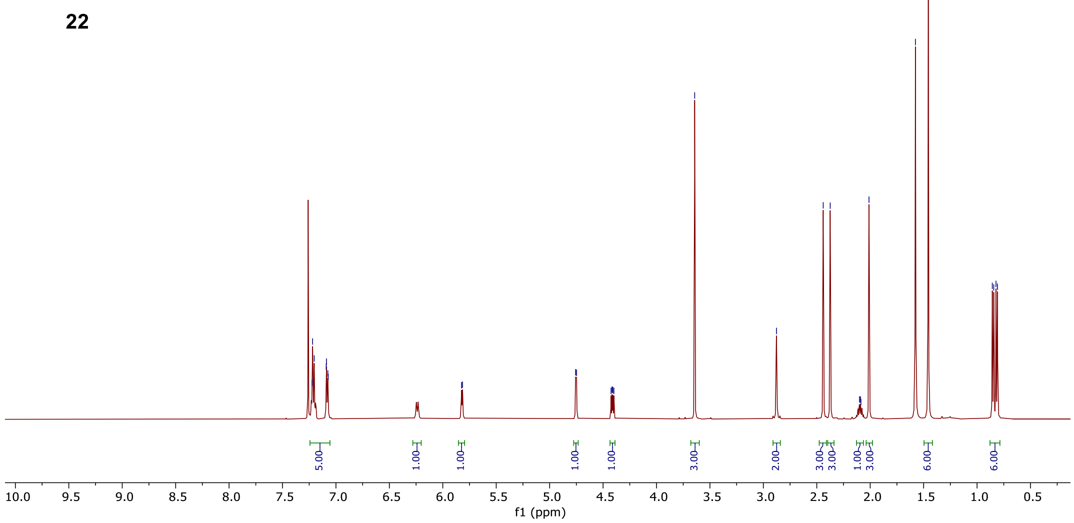

^{13}C-NMR 22

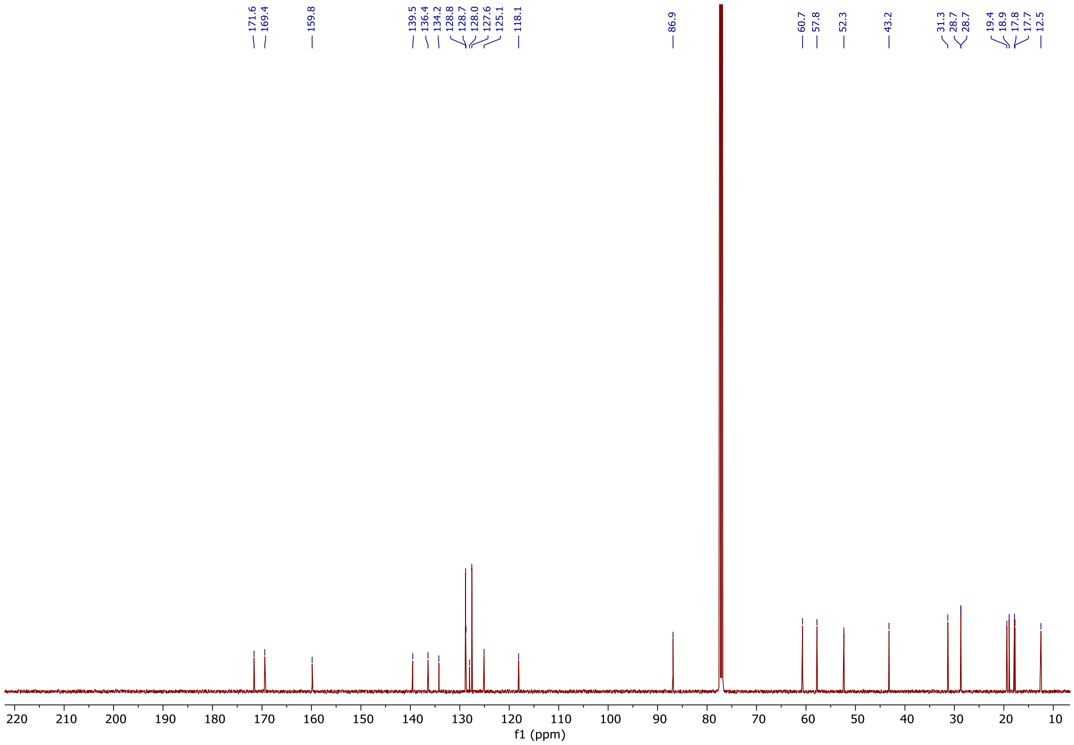

4.10 ^{1}H-NMR 23

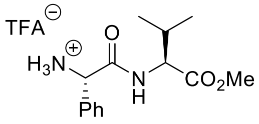

23

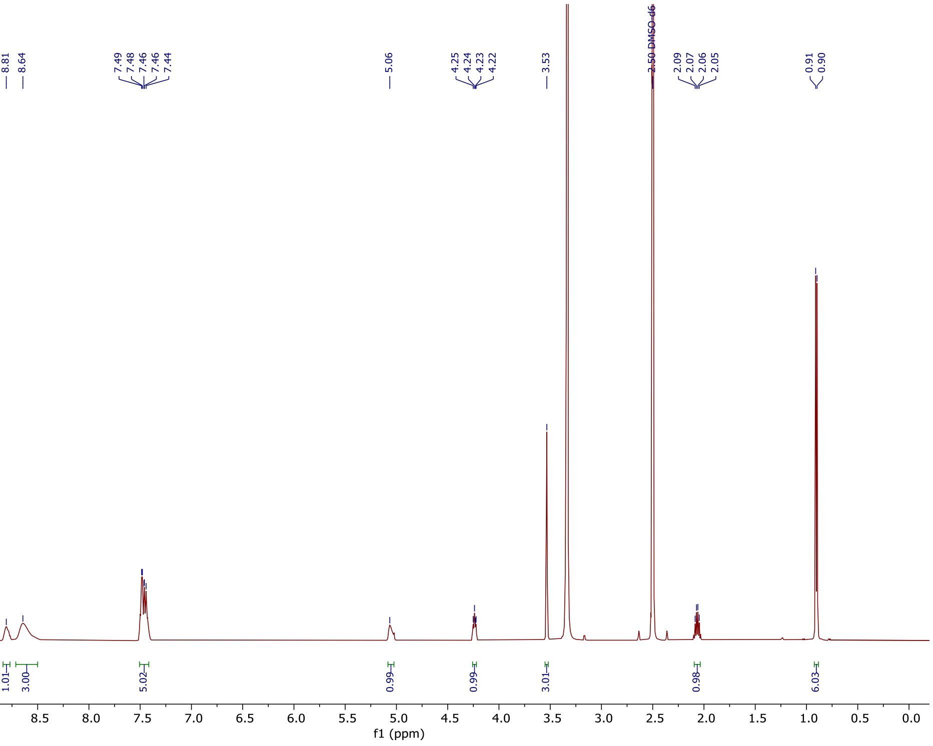

^{13}C-NMR 23

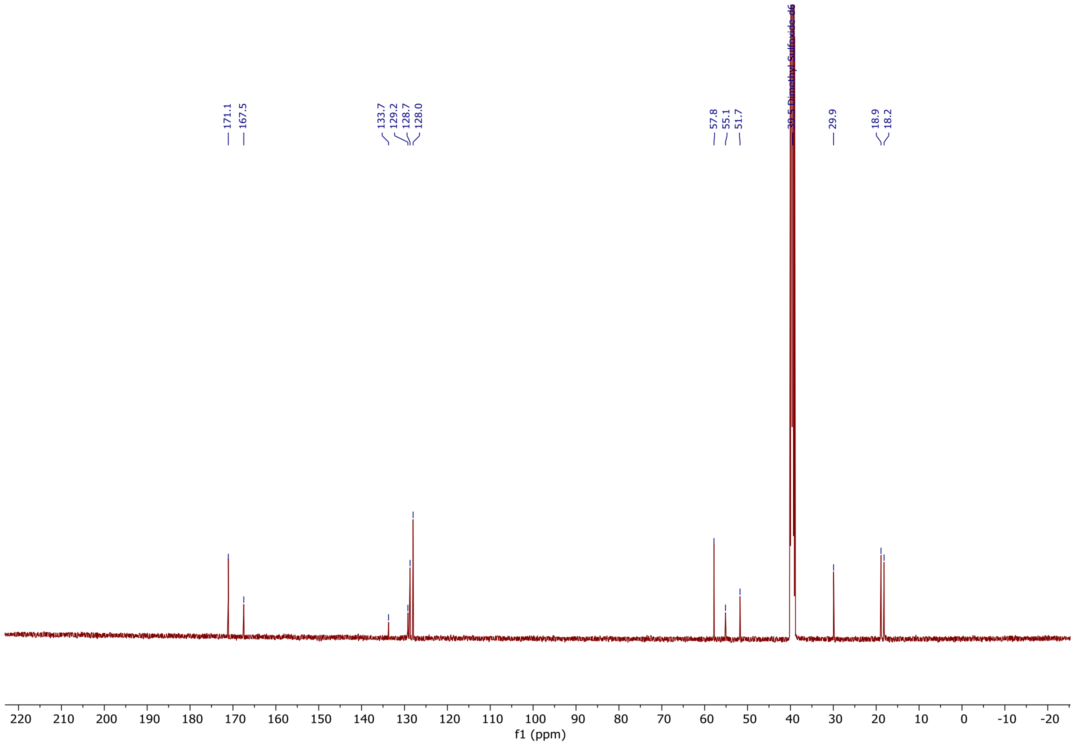

4.11 ^{1}H-NMR 25a

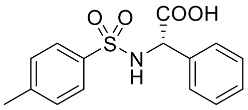

25a

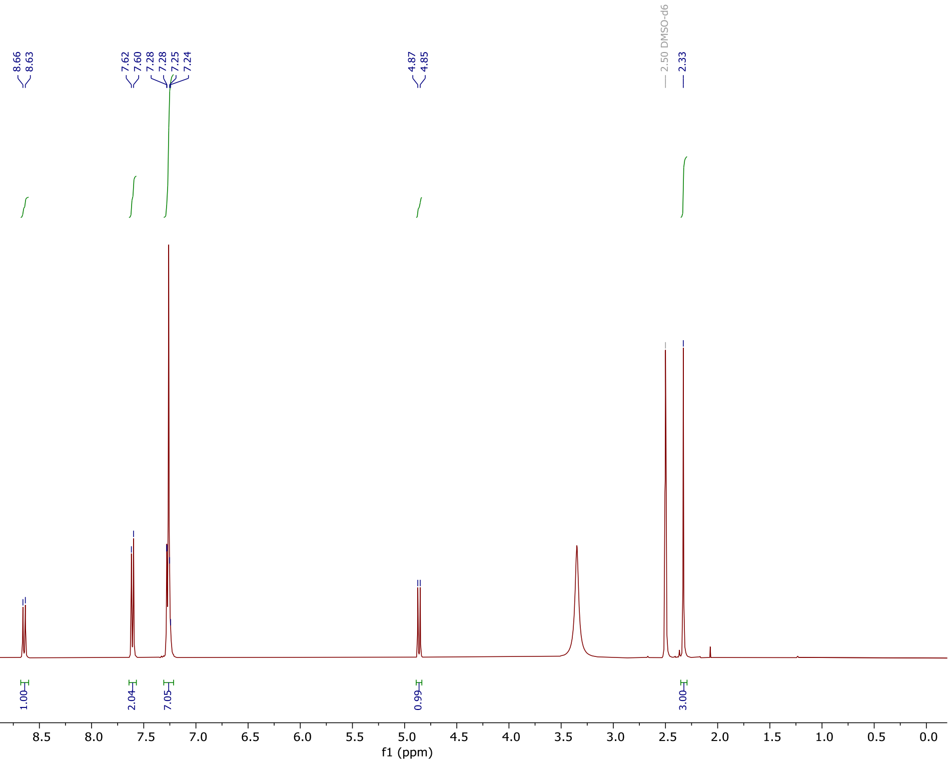

^{13}C-NMR 25a

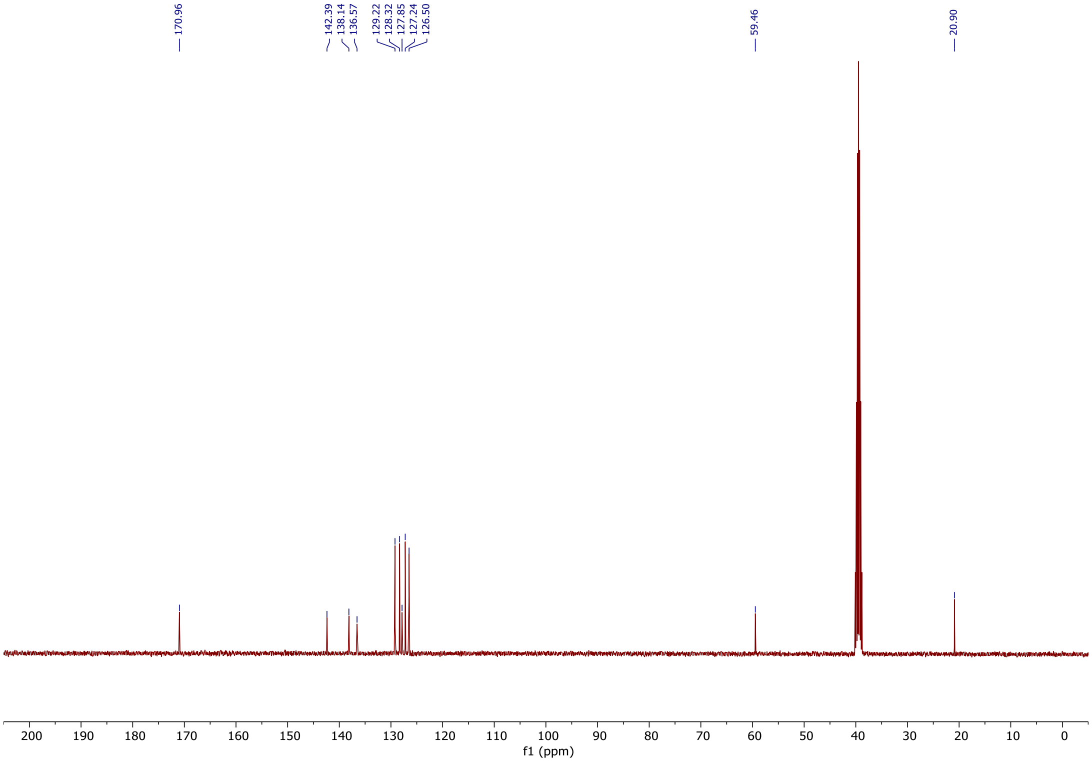

4.12 ¹H-NMR 27a

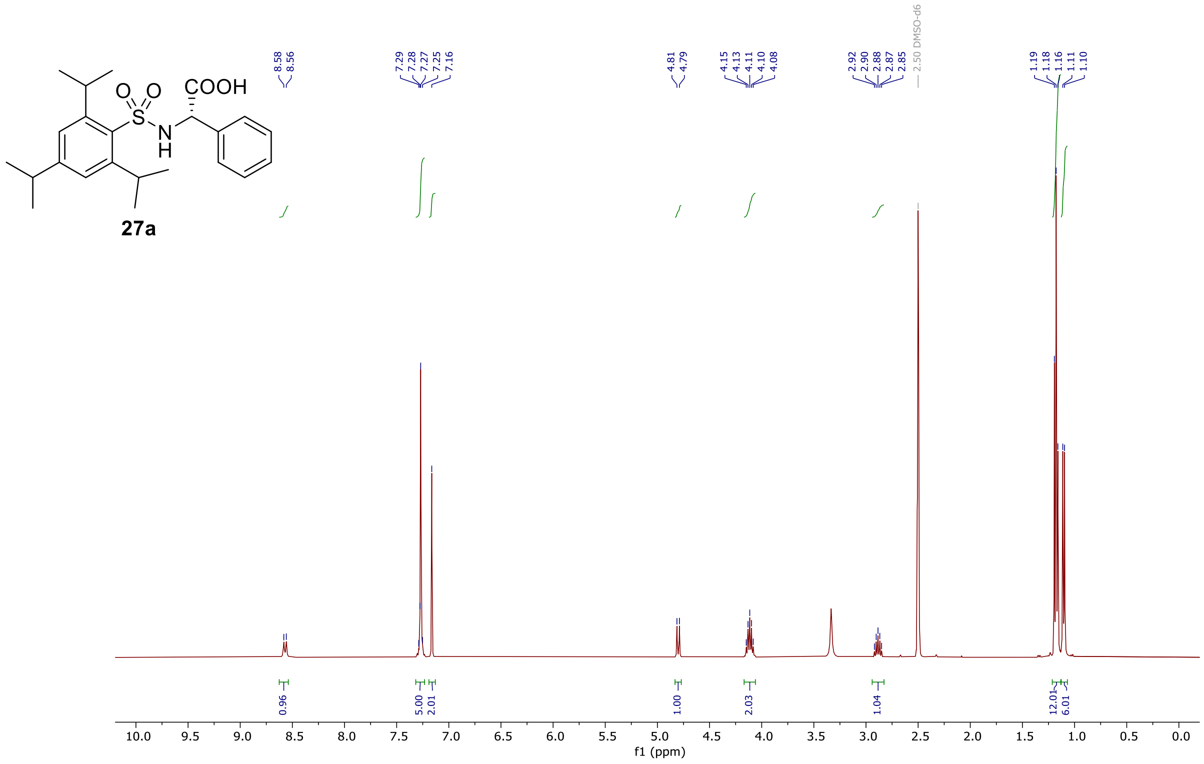

5 Xray Data

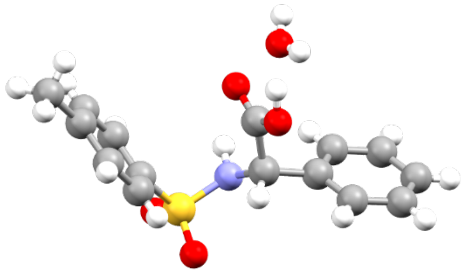

Table 1. Crystal data and structure refinement for **25a**. Displacement ellipsoids are shown at the 50% probability level. The crystal data are equivalent to the according literature. [3]

Identification code	TOVXEJ 1022467	
Empirical formula	C15 H17 N O5 S	
Formula weight	323.35	
Temperature	150(2) K	
Wavelength	1.54184 Å	
Crystal system	Orthorhombic	
Space group	P 21 21 21	
Unit cell dimensions	a = 5.59560(10) Å	α = 90°.
	b = 15.9422(3) Å	β = 90°.
	c = 17.1305(4) Å	γ = 90°.
Volume	1528.15(5) $Å^3$	
Z	4	
Density (calculated)	1.405 Mg/m^3	
Absorption coefficient	2.100 mm^{-1}	
F(000)	680	
Crystal size	0.450 x 0.080 x 0.080 mm^3	
Theta range for data collection	3.788 to 62.601°.	
Index ranges	-6<=h<=6, -11<=k<=18, -18<=l<=19	
Reflections collected	7275	
Independent reflections	2441 [R(int) = 0.0402]	
Completeness to theta = 62.601°	100.1 %	

Absorption correction	Analytical
Max. and min. transmission	0.837 and 0.436
Refinement method	Full-matrix least-squares on F^2
Data / restraints / parameters	2441 / 3 / 209
Goodness-of-fit on F^2	1.077
Final R indices [I>2sigma(I)]	R1 = 0.0463, wR2 = 0.1219
R indices (all data)	R1 = 0.0476, wR2 = 0.1233
Absolute structure parameter	-0.008(19)
Extinction coefficient	n/a
Largest diff. peak and hole	0.425 and -0.472 e.Å^{-3}

6 References

[1] Carpino, Louis A., Hitesh Shroff, Salvatore A. Triolo, El-Sayed M.E. Mansour, Holger Wenschuh and Fernando Albericio. 1993. "The 2,2,4,6,7-pentamethyldihydrobenzofuran-5-sulfonyl group (Pbf) as arginine side chain protectant." *Tetrahedron Letters*, 34, 7829-7832. https://doi.org/10.1016/S0040-4039(00)61487-9

[2] Denmark, Scott E., Robert A. Stavenger, Anne-Marie Faucher and James P Edwards. 1997. "Cyclopropanation with Diazomethane and Bis(oxazoline)palladium(II) Complexes". *The Journal of Organic Chemistry*, 62, 3375-3389. https://doi.org/10.1021/jo970044z

[3] Duarte-Hernández, Angélica M., Rosalinda Contreras, Galdina V. Suárez-Moreno, Pedro Montes-Tolentino, Iris Ramos-García, Felipe J. González and Angelina Flores-Parra. 2015. "(S) 2-phenyl-2-(*p*-tolylsulfonylamino)acetic acid. Structure, acidity and its alkali carboxylates." *Journal of Molecular Structure*, 1084, 135-147. https://doi.org/10.1016/j.molstruc.2014.11.057
